# Focal adhesion proteins confer smooth muscle anoikis resistance and protection against aortic aneurysm and dissection

**DOI:** 10.1172/jci.insight.195291

**Published:** 2026-03-24

**Authors:** Zhenyuan Zhu, Mingjun Liu, Jianxin Wei, Deepa Suryanarayan, Parya Behzadi, Robert Edgar, Julie A. Phillippi, Cynthia St. Hilaire, Cristina Espinosa-Diez, Delphine Gomez

**Affiliations:** 1 Pittsburgh Heart, Lung, Blood, and Vascular Medicine Institute, University of Pittsburgh, Pittsburgh, Pennsylvania, USA.; 2 School of Medicine, Tsinghua University, Beijing, China.; 3 Department of Cardiothoracic Surgery,; 4 Department of Bioengineering, Swanson School of Engineering, and; 5 Department of Medicine, Division of Cardiology, University of Pittsburgh, Pittsburgh, Pennsylvania, USA.; 6 Center for Molecular Medicine and Genetics, Department of Physiology, Wayne State School of Medicine, Detroit, Michigan, USA.

**Keywords:** Cell biology, Vascular biology, Apoptosis survival pathways, Cytoskeleton, Extracellular matrix

## Abstract

Thoracic aortic aneurysm and dissection (TAAD) involves a progressive dilation of the aortic wall associated with degradation of the extracellular matrix (ECM), cystic medial degeneration, smooth muscle cell (SMC) dysfunction, and rarefaction. TAAD etiology and pathogenesis suggest that alteration of mechanical force propagation may contribute to SMC dysfunction. This study aims to determine the role of SMC focal adhesion proteins, which are key components of force transmission, in TAAD pathogenesis. scRNA-seq analysis of human TAA aortas showed reduced expression of intracellular focal adhesion components, including *PTK2* (FAK), *VCL*, *ILK*, and *TES* transcripts, in SMCs. Additionally, protein levels of FAK, ILK, and VCL were decreased in the aorta of patients with TAA. SMC-specific *Ptk2*, *Vcl*, and *Ilk* KO mice treated with β-aminopropionitrile (BAPN) exhibited increased mortality, aortic dilation, ECM breakdown, and SMC loss. Mechanistically, knocking down FAK, ILK, and VCL exacerbated gliotoxin-induced SMC anoikis, whereas overexpressing full-length WT and dead-kinase FAK conferred resistance to apoptosis and cell detachment, indicating that FAK’s protective effects depend on its expression rather than its enzymatic activity. Inhibition of FAK kinase activity did not affect SMC apoptosis in vitro or aortic dilation in vivo. Our findings demonstrate that the expression of focal adhesion proteins protects against TAAD progression and SMC anoikis independently of FAK kinase activity.

## Abstract 2

Focal adhesion proteins help prevent aortic wall weakening by supporting smooth muscle cell survival and resisting anoikis, offering new insight into aneurysm progression and potential treatments.

## Graphical abstract

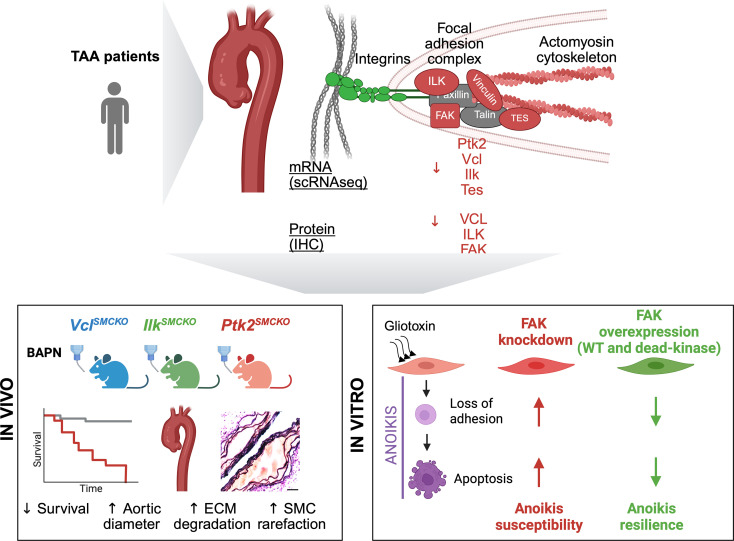

## Introduction

Thoracic aortic aneurysm and dissection (TAAD) is a life-threatening condition characterized by the progressive weakening and dilation of the aorta, affecting approximately 10 in 100,000 individuals annually (1). Various factors are associated with TAAD development, such as age, the presence of a congenital bicuspid aortic valve, and syndromic (e.g., Marfan Syndrome [MFS]), and nonsyndromic genetic variations (2, 3). TAAD exhibits common pathological features, including cystic medial degeneration, elastin fragmentation, aortic medial SMC dysfunction, and SMC rarefaction in advanced stages, regardless of underlying causes (2, 4). SMCs have significant plasticity, allowing them to transition from a differentiated, quiescent, and contractile phenotype to context-dependent noncontractile phenotypes associated with the acquisition of functions such as proliferation, migration, ECM synthesis, and remodeling (5). Seminal studies have identified SMC phenotypic modulation as an early event in aneurysm formation, marked by decreased expression of SMC contractile genes (e.g., ACTA2, MYH11, TAGLN) and increased expression of ECM remodeling markers such as matrix metalloproteinases in both human and experimental models of TAAD (6). Single-cell RNA-seq (scRNA-seq) in MFS mouse models and patients with TAA uncovered a complex and dynamic SMC phenotypic modulation, supporting a model where, as the disease progresses, SMCs shift from a primarily contractile phenotype to alternative states that contribute to ECM remodeling and medial degeneration (7, 8).

ECM composition influences mechanotransduction pathways and the intracellular integration of mechanical forces. Loss-of-function mutations and genetic variants affecting genes encoding mechanotransduction components have been associated with TAAD occurrence in humans, including ECM (*FBN1*), focal adhesion complex (*TES*), and contractile and cytoskeletal proteins (*ACTA*, *MYH11*) (9–14). The focal adhesion complex bridges the ECM with the contractile apparatus within SMC, ensuring cell adherence, force propagation, and cellular adaptation to mechanical strain. On the one hand, studies reported the upregulation of focal adhesion–related pathways in aneurysmal SMC based on single-cell transcriptomic analysis (7, 8). For example, integrin αV is upregulated in patients with MFS and mouse models (15, 16). Integrin αV blockage reduced aortic dilation and preserved SMC contractile phenotype (16). MFS-derived iPSCs show increased integrin αV activation, promoting a proliferative, migratory SMC phenotype reflective of early TAA pathology (17). On the other hand, evidence indicates that other key members of the focal adhesion complex are downregulated in TAA. Decreased Vinculin (VCL) expression has been reported in aortic sections of patients with TAAD and TADD mouse models, mirroring the overall reduction of contractile proteins and medial degeneration (18, 19). Missense mutations in the focal adhesion scaffold protein Testin (TES) impaired SMC contractile gene expression and caused aortic aneurysm (13). Integrin-Linked Kinase (ILK) KO in SMC and SMC progenitors resulted in severe cardiovascular defects during embryonic development, including aortic dilation and tunica media disorganization (20, 21). These findings suggest focal adhesion complex imbalance could promote TAA progression (22, 23).

Focal adhesion kinase (FAK) plays a versatile role in regulating SMC phenotypes and functions. Firstly, FAK functions as a structural scaffold within the focal adhesion complex, localizing constitutively to adhesions and organizing key proteins, such as paxillin, talin, and integrins, through its multidomain architecture (FERM, proline-rich motifs, FAT domain). Loss of FAK or disruption of its interaction domains alters focal adhesion morphology and dynamics, and many structural aspects of adhesion assembly can be rescued by kinase-dead FAK, indicating a nonenzymatic structural role (24–27). Secondly, FAK activation and cytoplasmic localization promote SMC proliferation and vascular injury-induced neointima formation, whereas catalytically inactive FAK localizes in the nucleus to maintain SMC quiescence and contractility (28–30). In Marfan-derived iPSCs, the aforementioned proproliferative effects of integrin αV are associated with FAK activation (17). Thirdly, FAK influences cell survival. In cancer, FAK suppression exacerbated anoikis, a distinct form of programmed cell death due to loss of cell adhesion (31, 32). These observations are directly related to TAAD pathophysiology, as increased ECM degradation and pericellular proteolytic and fibrinolytic activity negatively affect SMC adhesion to the ECM and trigger SMC apoptosis (33, 34). It has been postulated that SMC rarefaction due to apoptosis observed in TAAD (35, 36) results from anoikis induced by advanced matrix degeneration and disengagement of ECM/integrin interactions (37, 38). However, the relative contribution of SMC-derived focal adhesion protein complexes, such as VCL, ILK, or FAK on TAAD progression and SMC rarefaction through anoikis, has not been comprehensively investigated.

Our studies investigate the role of VCL, ILK, and FAK, which were found to be downregulated in human TAA. The effect of SMC-specific KO of these focal adhesion components was evaluated in an experimental model of TAAD induced by Lysyl oxidase inhibitor, β-aminopropionitrile (BAPN), treatment (36, 37). Additionally, we sought to uncover how modulation of FAK expression influenced SMC adhesion and apoptosis in an in vitro model of anoikis. We found that decreased expression of focal adhesion proteins, VCL, ILK, and FAK caused exacerbation of BAPN-induced TAAD progression, related mortality, and SMC rarefaction due to apoptosis. FAK expression provided SMC resilience against detachment-induced cell death, independently of its kinase activity.

## Results

### Dysregulation of focal adhesion signaling pathways in TAA.

**Figure 1:**
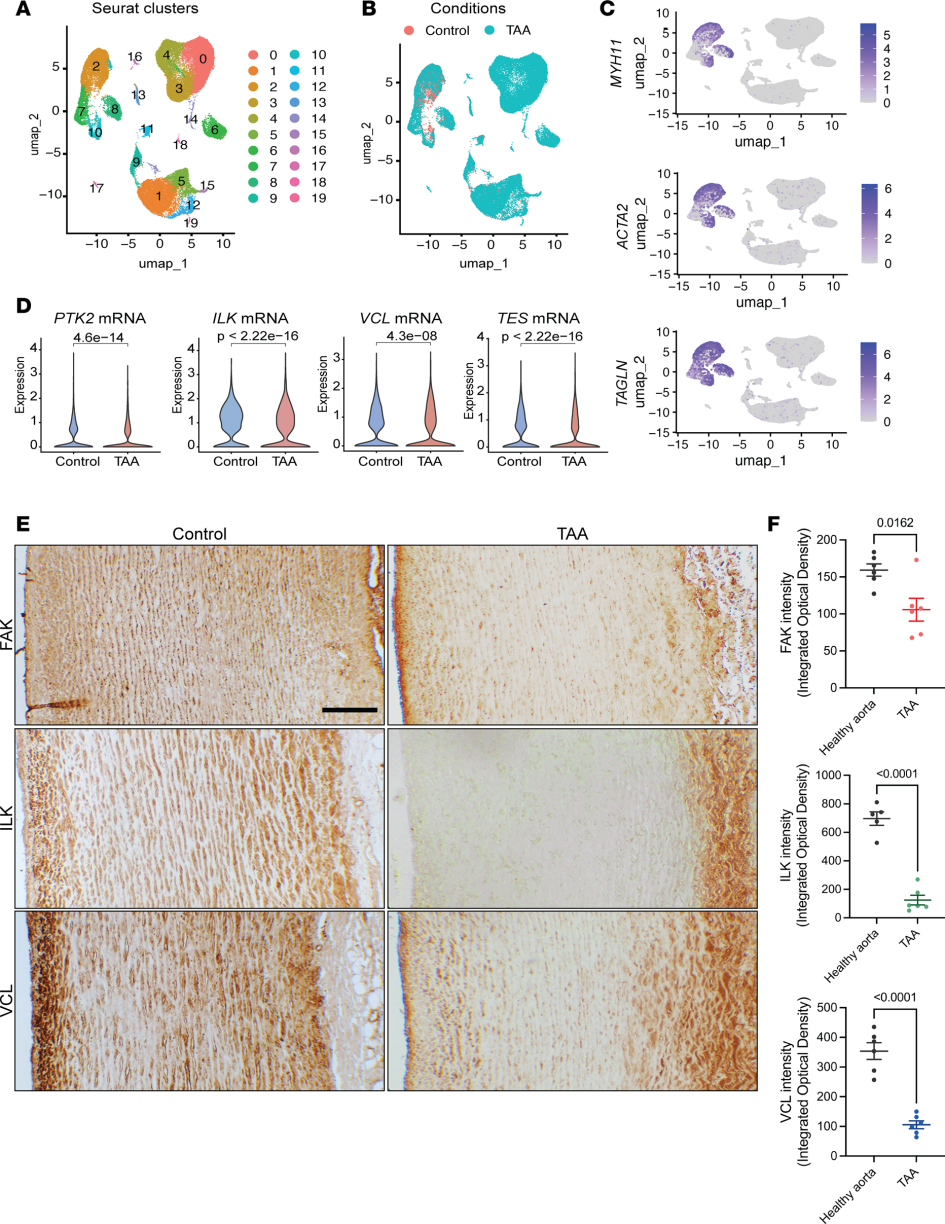
Reduced expression of key focal adhesion complex components in human thoracic aortic aneurysm. (**A**) UMAP dimensionality reduction plot of all aortic cell populations derived from TAA and control aortas (data from GSE155468). (**B**) UMAP representation showing cell clustering by condition. (**C**) UMAP plots displaying *MYH11*, *ACTA2*, and *TAGLN* expression in aortic cell clusters. Clusters 2, 7, 8, and 10 expressed these markers and were identified as SMC. (**D**) Violin plots illustrating the expression of *PTK2*, *ILK*, *VCL*, and *TES* transcripts. A significant downregulation of these transcripts was observed in TAA compared with controls. Wilcoxon test. (**E**) Representative micrographs showing FAK, ILK, and VCL protein expression in the aortas of patients with TAA and healthy controls. Scale bar: 100 μm. (**F**) Quantification of IHC staining intensity. Student’s *t* test.

Recent scRNA-seq uncovered SMC phenotypic modulation and the dysregulation of central biological processes and signaling pathways in patients with TAA (8, 39, 40). We performed a secondary analysis of a publicly available scRNA-seq data from TAA and healthy control aortas (GSE155468). SMC clusters identified based on the expression of lineage-specific markers (*MYH11*, *ACTA2*, *TAGLN*) were found in healthy and TAA aortas (Figure 1, A–C). Importantly, the levels of *PTK2* (FAK), *ILK*, *VCL*, and *TES* (Testin) mRNAs in the SMC subclusters were downregulated in TAA compared with healthy controls (Figure 1D). Similar results were observed in the aorta of MFS mice (*Fbn1^C1041G/+^*), which develop aortic aneurysms (GSE153534) (7). *Fbn1^C1041G/+^* aortas presented with an increased proportion of transcriptionally modulated smooth muscle cells (modSMCs) exhibiting reduced expression of contractile genes (*Acta2*, *Myh11*, *Tagln*, and *Mylk*), alongside elevated *Fn1* (Fibronectin 1) transcript counts (Supplemental Figure 1, A–D; supplemental material available online with this article; https://doi.org/10.1172/jci.insight.195291DS1). KEGG analysis of differentially expressed genes between modSMC and contractile SMC revealed the downregulation of the focal adhesion pathway, which was associated with decreased transcript levels of focal adhesion complex proteins (*Ilk*, *Limd1*, *Lims1*, *Lims2*, *Tes*, *Vcl*) (Supplemental Figure 1, E and F). These data indicate that several of the key components of the focal adhesion complex are transcriptionally downregulated in aneurysmal vessels in human and mice. We investigated FAK, ILK, and VCL protein expression in human TAA and healthy control aortic specimens (Supplemental Figure 2). IHC revealed marked downregulation of FAK, VCL, and ILK in the aortic walls of patients with TAA compared with controls (Figure 1, E and F). Interestingly, heterogeneity in FAK, VCL, and ILK expression patterns was observed in the medial layer of aneurysmal aorta with marked downregulation in the two-thirds proximal to the intima (Supplemental Figure 3). Medial areas adjacent to the adventitia maintained high expression of FAK, VCL, and ILK. Together, these findings provide evidence of reduced expression of key components of the focal adhesion complex in aneurysmal SMCs.

### FAK deletion in SMC worsens TAAD development.

**Figure 2:**
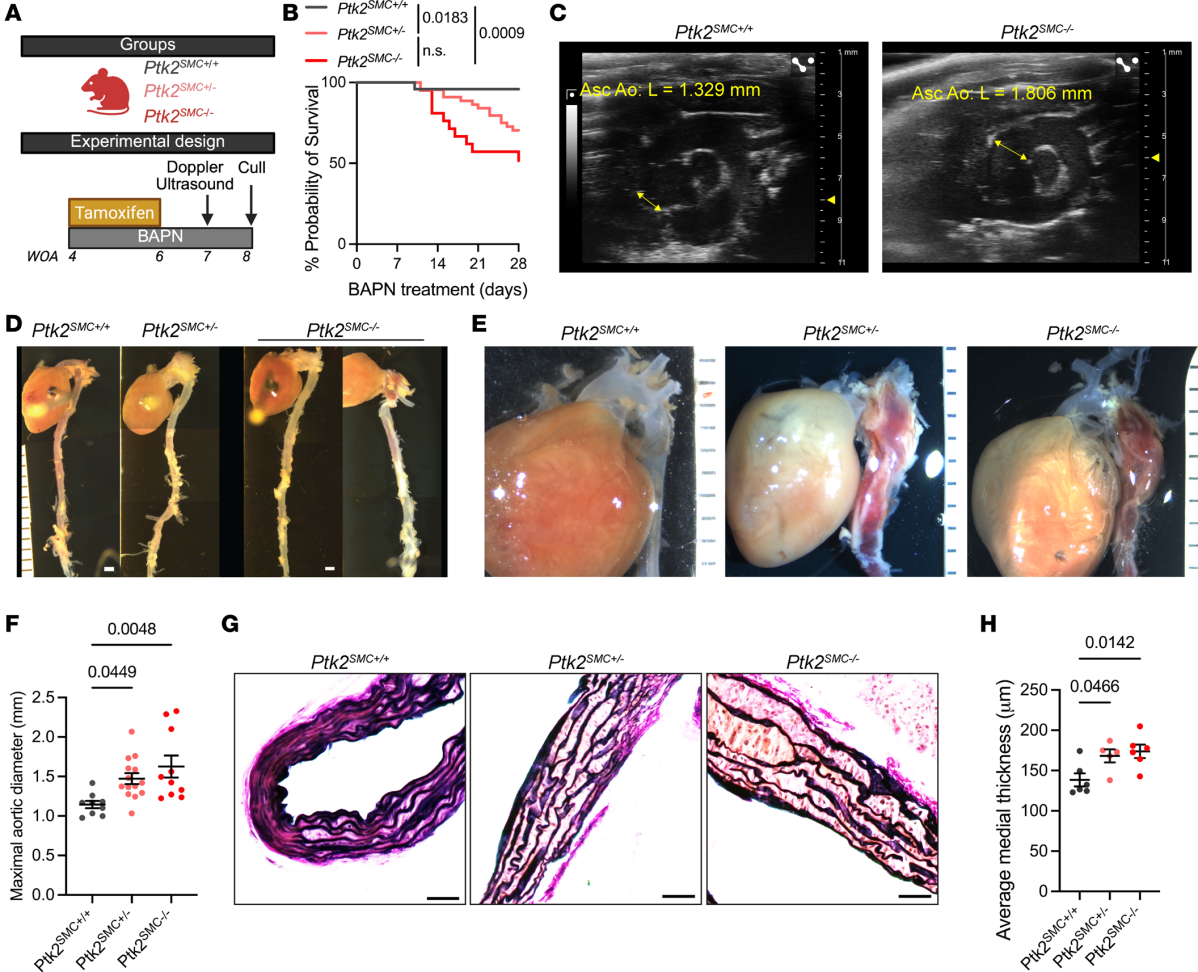
*Ptk2* deletion in SMC worsens TAA development in a gene dosage–dependent manner. (**A**) Schematic of *Ptk2^SMC–/–^* mouse cohort alongside the tamoxifen and BAPN treatment timeline in weeks of age (WOA). (**B**) Survival probability of *Ptk2^SMC+/+^*, *Ptk2^SMC+/–^*, and *Ptk2^SMC–/–^* mice during 28 days of BAPN treatment. Statistical significance between groups was determined using the Kaplan-Meier test, with significance defined as *P* < 0.05. (**C**) Representative ultrasound micrographs of *Ptk2^SMC+/+^* and *Ptk2^SMC–/–^* at 28 days post BAPN. (**D**) Representative images of the aortas of *Ptk2^SMC+/+^*, *Ptk2^SMC+/–^*, and *Ptk2^SMC–/–^* mice after 28 days of BAPN treatment. Scale bar: 1 mm. (**E**) Examples of aortic dissections in *Ptk2^SMC+/–^*, and *Ptk2^SMC–/–^* mice. Scale bar: 1 mm. (**F**) Maximum aortic diameter measurements for *Ptk2^SMC+/+^*, *Ptk2^SMC+/–^*, and *Ptk2^SMC–/–^* *groups*. One-way ANOVA. (**G**) Representative micrographs of Verhoeff-Van Gieson (VVG) elastin staining. Scale bar: 100 μm. (**H**) Quantification of the average aortic medial thickness. Student’s *t* test.

**Figure 3:**
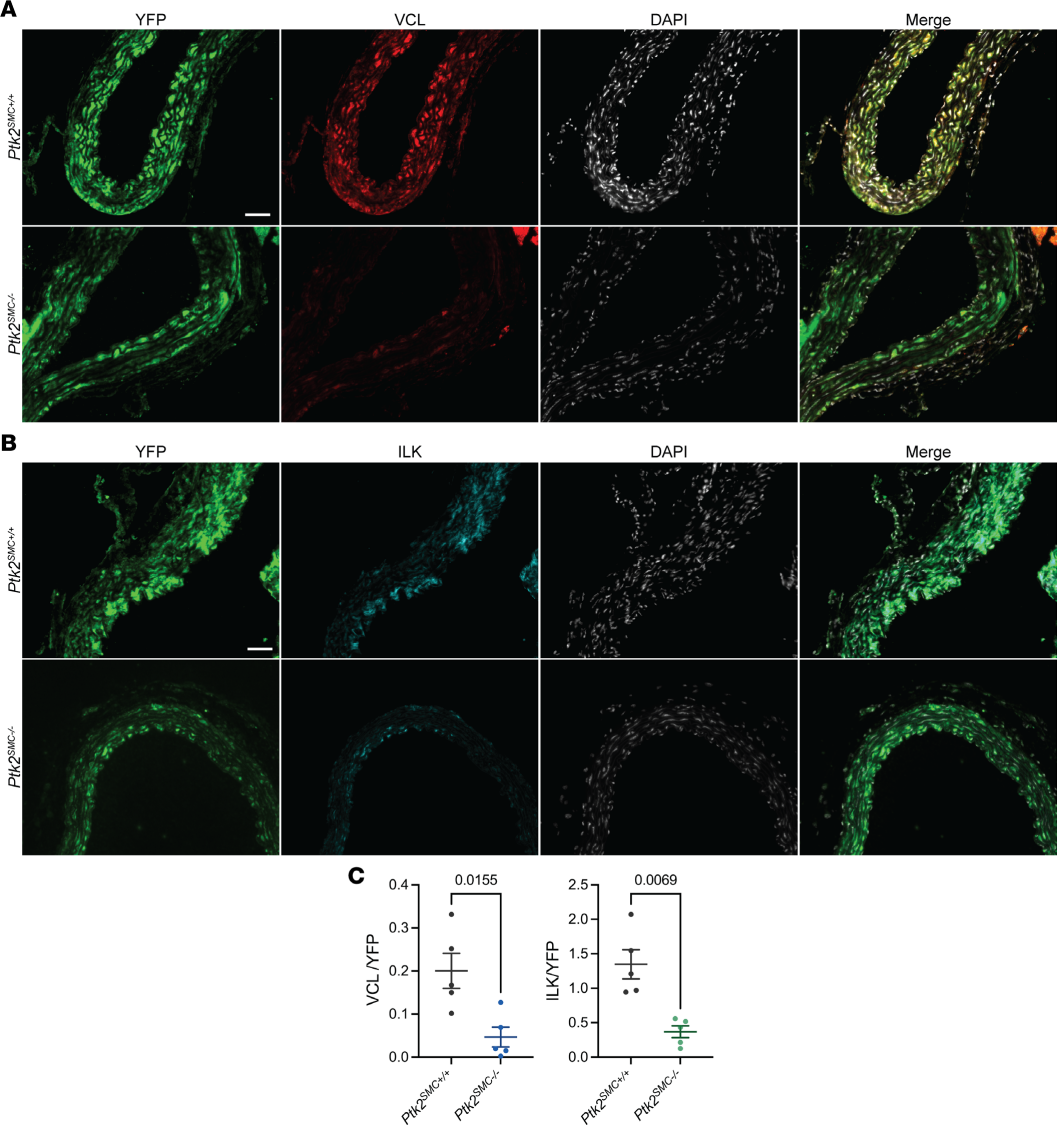
*Ptk2* deletion in SMC promotes the downregulation of VCL and ILK in vivo. (**A**) Representative micrographs of YFP (Green), VCL (red), and DAPI (white) staining in aortic cross-sections. Scale bar: 100 μm. (**B**) Representative micrographs of YFP (green), ILK (cyan), and DAPI (white) staining in aortic cross-sections. Scale bar: 100 μm. (**C**) Quantification of VCL^+^/YFP^+^ and ILK^+^/YFP^+^ medial area. Student’s *t* tests.

While modulation of integrin signaling has been evaluated in the context of aortic aneurysm (16), there remains a substantial gap in directly assessing the role of focal adhesion complex components in the development and progression of aortic dilation and SMC dysfunction. FAK, encoded by the *PTK2* gene, is a central component of the focal adhesion complex and regulates SMC differentiation, proliferation, and survival (28–30). We generated SMC lineage-tracing and specific *Ptk2*-KO mice, in which tamoxifen induced excision of *Ptk2* Exon 3 and YFP expression to enable rigorous fate mapping (Supplemental Figure 4). *Ptk2^SMCflox^* mice were treated with BAPN for 28 days starting at 4 weeks of age (Figure 2A). BAPN is an irreversible inhibitor of Lysyl oxidase and prevents ECM maturation by disrupting collagen and elastin cross-linking. BAPN treatment induces both aortic dilation and dissection (Supplemental Figure 5) (41). Homozygous *Ptk2^SMC–/–^* and heterozygous *Ptk2^SMC+/–^* mice presented with increased premature death compared with WT littermates at 28 days of BAPN treatment (Figure 2B). This decrease in survival was associated with enhanced aortic dilation of the thoracic aorta, as observed pre- (Doppler-ultrasound) and postmortem (Figure 2, C and D). Aortic dissections were also observed in *Ptk2^SMC–/–^* and *Ptk2^SMC+/–^* mice (Figure 2E). Consistent with these observations, a significant increase in the aortic diameter was measured in both *Ptk2^SMC–/–^* and *Ptk2^SMC+/–^* compared with *Ptk2^SMC+/+^* mice (Figure 2F). Loss of FAK expression in SMC promoted severe ECM disorganization and medial thickening (Figure 2, G and H). Concomitantly, a decrease in contractile marker (ACTA2) intensity, as well as a significant reduction in SMC tracker YFP positivity, was observed in *Ptk2^SMC–/–^* aortas (Supplemental Figure 6). The latter strongly suggests SMC loss, consistent with that observed in human late-stage TAAD. Finally, we evaluated the effect of FAK depletion on VCL and ILK expression. Remarkably, *Ptk2* KO in SMC was associated with a reduced expression of both VCL and ILK in YFP^+^ cells (Figure 3, A–C). These results provide strong evidence that loss or reduction of FAK expression in SMCs has deleterious effects on TAAD pathological features, aggravating aortic dilation and SMC rarefaction and causing downregulation of VCL and ILK.

### Focal adhesion proteins VCL and ILK protect against TAAD progression and associated mortality.

**Figure 4:**
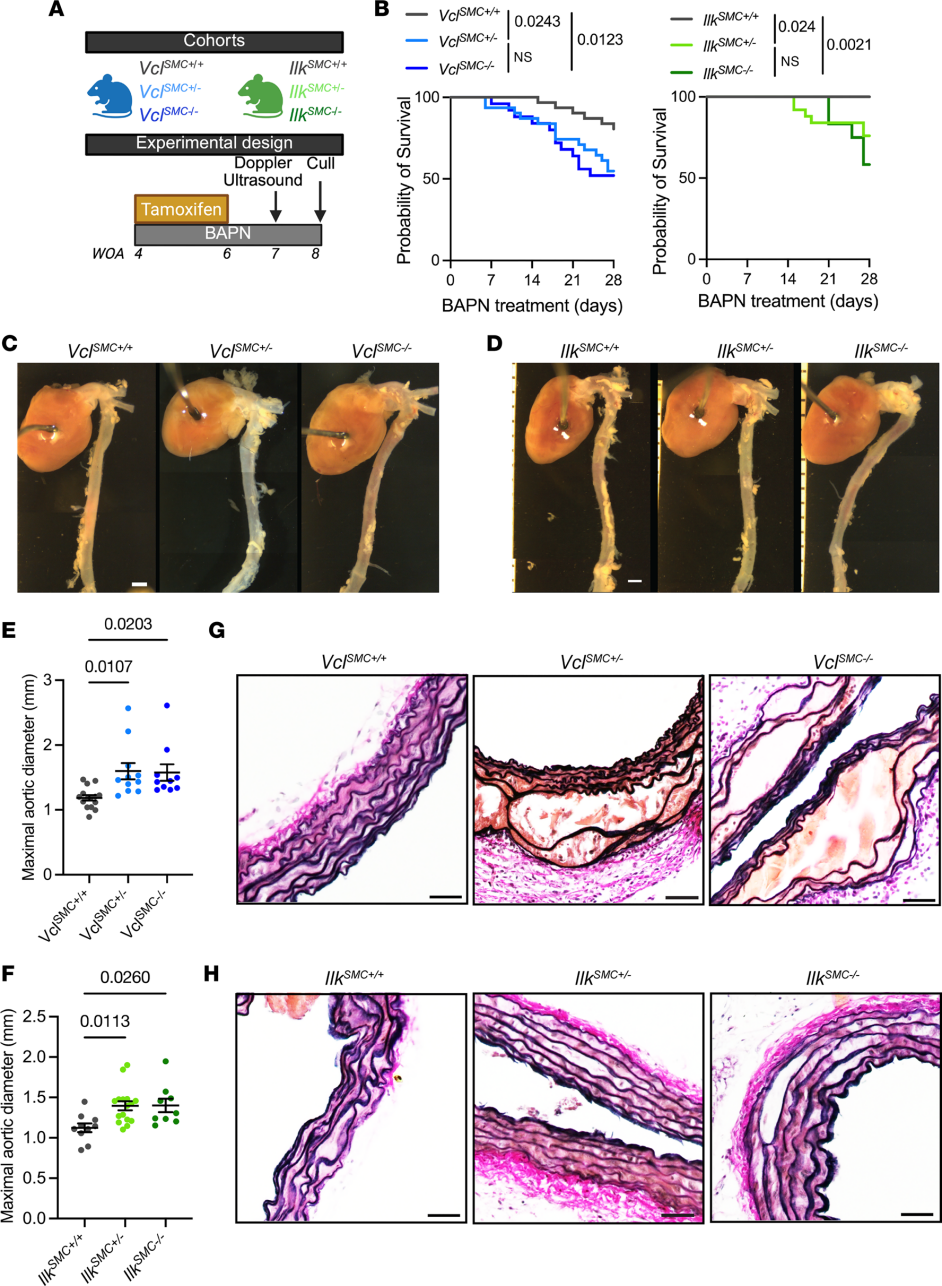
SMC-specific loss of VCL and ILK exacerbates thoracic aortic aneurysm in mice. (**A**) Schematic summarizing the experimental cohorts (*Vcl^SMCflox^* in blue and *Ilk^SMCflox^* in green) and experimental design consisting of treatment with tamoxifen and BAPN in 3- to 4-week-old mice. Mice are treated with BAPN for 4 weeks prior to euthanasia and aorta collection. (**B**) Survival curve in *Vcl^SMCflox^* and *Ilk^SMCflox^* mouse cohorts during 28 days of BAPN treatment. Statistical significance between groups was determined using Kaplan-Meier test, with significance defined as *P* < 0.05. (**C** and **D**) Representative images of the aortas of the *Vcl^SMC+/+^*, *Vcl^SMC+/–^*, and *Vcl^SMC–/–^* mice (**C**) and the *Ilk^SMC+/+^*, *Ilk^SMC+/–^*, and *Ilk^SMC–/–^* mice (**D**) after 28 days of BAPN treatment. Scale bar: 1 mm. (**E**) Maximal diameter of *Vcl^SMC+/+^*, *Vcl^SMC+/–^*, and *Vcl^SMC–/–^* aortas at 28 days. One-way ANOVA. (**F**) Maximal diameter of *Ilk^SMC+/+^*, *Ilk^SMC+/–^*, and *Ilk^SMC–/–^* aortas at 28 days. One-way ANOVA. (**G**) Representative micrographs of Verhoeff-Van Gieson (VVG) elastin staining in *Vcl^SMC+/+^*, *Vcl^SMC+/–^*, *Vcl^SMC–/–^* aortas. Scale bar: 50 μm. (**H**) Representative micrographs of Verhoeff-Van Gieson (VVG) elastin staining in *Ilk^SMC+/+^*, *Ilk^SMC+/–^*, and *Ilk^SMC–/–^* aortas. Scale bar: 50 μm.

We next investigated the effect of reduced expression of VCL and ILK, which was observed in human TAA aortas (Figure 1E) and exacerbated by FAK deficiency in SMC (Figure 3), on TAAD progression. We generated tamoxifen-inducible SMC fate mapping and specific Vcl (*Vcl^SMCflox^*) and Ilk (*Ilk^SMCflox^*) KO mouse lines (Supplemental Figure 4). These mice were treated with BAPN for 28 days (Figure 4A). *Vcl* KO (*Vcl^SMC–/–^*) and knockdown (KD) (*Vcl^SMC+/–^*) significantly reduced 28-day survival, with mortality approaching 50% (Figure 4B). Although less severe, *Ilk^SMC–/–^* and *Ilk^SMC+/–^* mice also exhibited significantly lower survival probabilities at 28 days of BAPN treatment (Figure 4B). In addition, *Vcl* and *Ilk* homozygote KO and heterozygote mice manifest exacerbated aortic dilation and higher aortic diameter compared with WT littermates (Figure 4, C–F). These results are similar to those observed in FAK-deficient mice. Histologically, ascending aortas from *Vcl^SMC–/–^* and *Ilk^SMC–/–^* mice displayed notable ECM disorganization, including elastic lamina breaks and increased medial thickness (Figure 4, G and H, and Supplemental Figure 7, A and B). SMC fate mapping through YFP staining showed that deletion of *Vcl^SMC–/–^* and *Ilk^SMC–/–^* induced a significant decrease in YFP^+^ area within the medial layer of the aorta, indicative of SMC rarefaction (Supplemental Figure 7, C–F). These results show that decreased expression of VCL and ILK in SMCs exacerbates aortic dilation, aneurysm formation, and mortality in BAPN-induced aortopathies, mediated by ECM disorganization and SMC rarefaction. These findings further support a causal relationship between decreased tissue levels of FAK, VCL, and ILK and aortic aneurysm and dissection progression.

### Loss of FAK, ILK, and VCL expression increases SMC sensitivity to anoikis.

**Figure 5:**
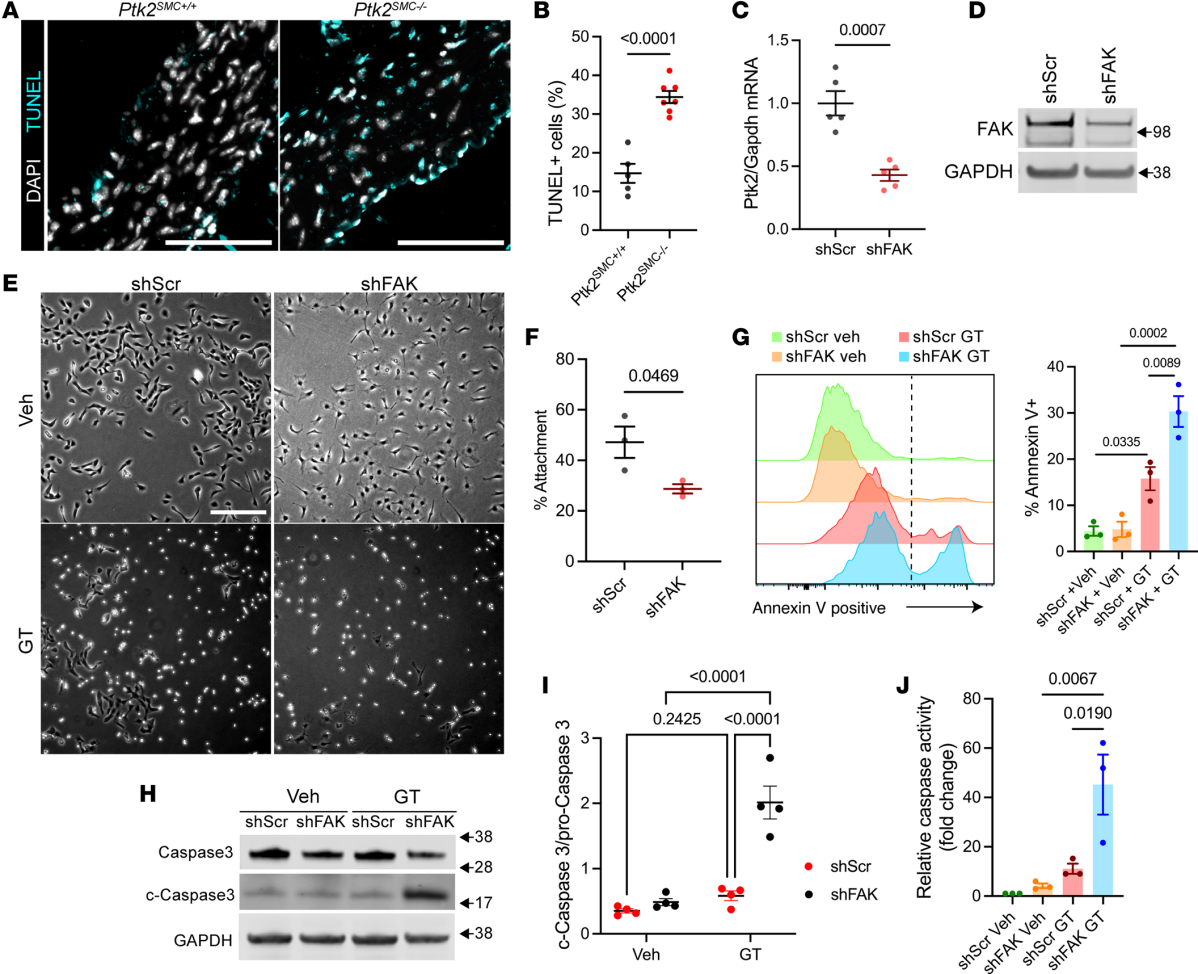
Loss of FAK expression increases SMC sensitivity to anoikis. (**A**) TUNEL staining in *Ptk2^SMC+/+^* and *Ptk2^SMC–/–^* aortas. DAPI: nuclear staining. Scale bar: 100 μm. (**B**) Quantification of medial TUNEL^+^ cells. Student’s *t* test. (**C**) qPCR analysis of Ptk2 transcript levels in SMC transduced with scrambled control shRNA (shScr) or gene Ptk2-targeting shRNA (shFAK). Data were normalized to GAPDH transcript expression. Student’s *t* test. *P < 0.05. (**D**) Western blot showing FAK protein expression in shFAK SMC compared with shScr SMC. The experiment was repeated 4 times, independently. (**E**) Representative micrographs of shFAK and shScr SMC treated with vehicle or gliotoxin for 6 hours. Scale bar: 200 μm. (**F**) Quantification of cell detachment in shFAK SMCs compared with shScr control. Data were presented as the percentage of attached cells after gliotoxin treatment relative to the vehicle-treated group. Student’s *t* test. (**G**) Annexin V expression by flow cytometry. Left: Representative flow cytometry histograms of Annexin V–Alexa Fluor 647 staining in shFAK and shScr SMCs treated with vehicle or Gliotoxin. Right: Quantification of Annexin V^+^ cells, shown as the percentage of total cells. One-way ANOVA. (**H**) Representative Western blot showing level of cleaved Caspase 3 and pro-Caspase 3. (**I**) Densitometric analysis of Pro-Caspase 3 and cleaved-Caspase 3 Western Blot. Results expressed as cleaved-Caspase 3/Pro-Caspase 3. One-way ANOVA. (**J**) Caspase 3/7 activity in shFAK and shScr SMCs treated with vehicle or gliotoxin. Fold change of luminescence relative to vehicle-treated shScr control. One-way ANOVA.

**Figure 6:**
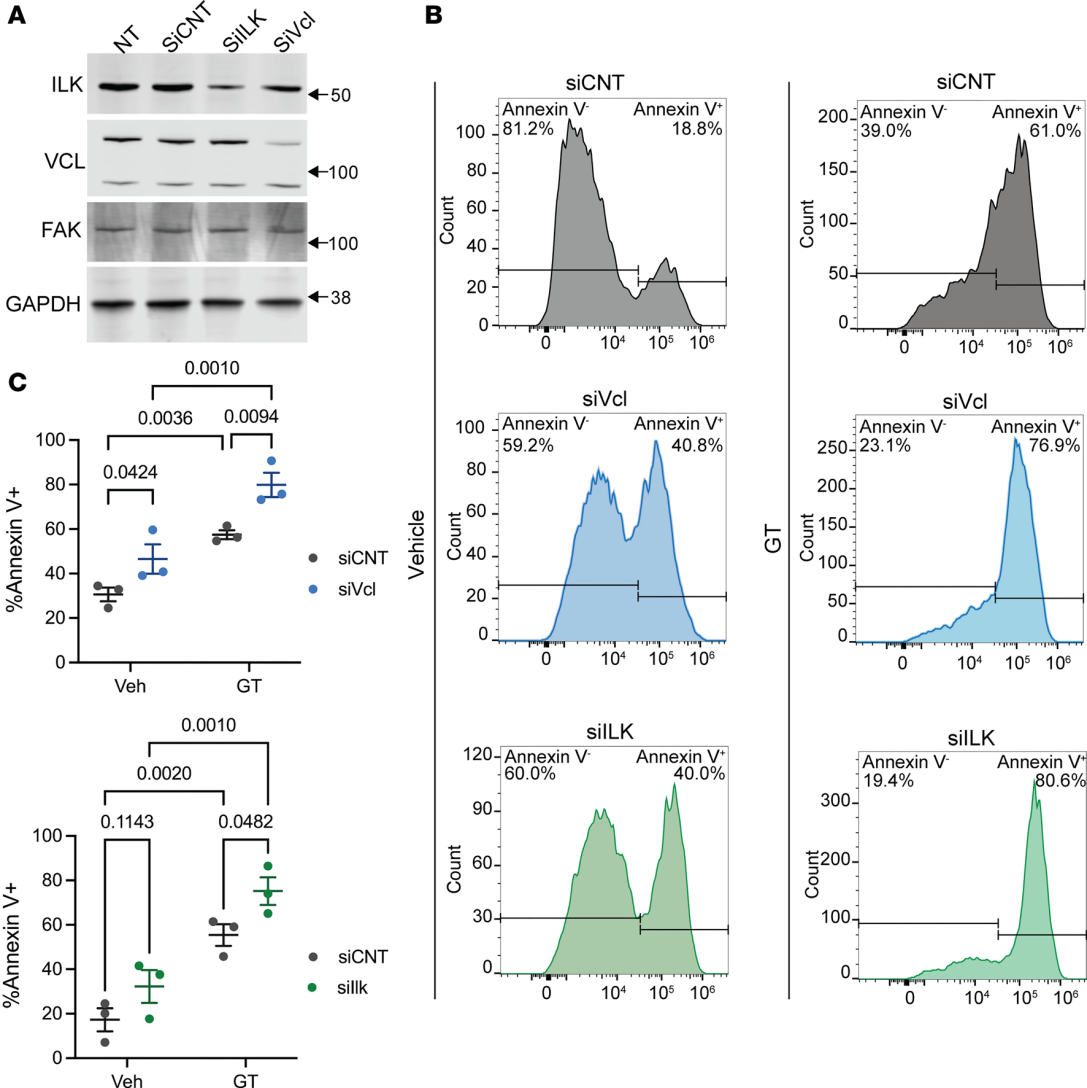
VCL and ILK knockdown potentiates gliotoxin-induced SMC apoptosis. (**A**) Representative Western blots showing the expression of ILK, VCL, and FAK in SMC treated with siILK or siVCL. GAPDH is used as a loading control. The experiment was conducted 3 times, independently. (**B**) Representative flow cytometry histograms of Annexin V–Alexa Fluor 647 staining in SMCs treated with siVCL or siILK, followed by incubation with vehicle or Gliotoxin (1 μM, 6 hours). (**C**) Quantification of Annexin V^+^ cells, shown as the percentage of total cells. One-way ANOVA.

We tested whether the decrease in SMC density resulted from increased cell death in *Ptk2^SMC–/–^* aortas. Medial cell death was increased by over 50% in *Ptk2^SMC–/–^* mice compared with WT littermates, as evidenced by TUNEL staining (Figure 5, A and B). Anoikis, or detachment-induced cell death, has been associated with SMC rarefaction in TAAD and is driven by disruption of the cell-ECM interactions (42). To investigate the effect of FAK downregulation on anoikis, we generated shRNA-induced FAK (shFAK) KD in SMC via lentivirus transduction, resulting in efficient reduction of FAK transcript and protein levels (Figure 5, C and D). A large body of research has associated FAK activation and SMC proliferation (28–30). As expected, shFAK was associated with decreased growth factor-mediated SMC proliferation (Supplemental Figure 8). Next, we treated cells with gliotoxin, an anoikis inducer, which triggers programmed cell death via cell detachment by inhibiting proper interaction between ECM and integrins (42). We confirmed this mechanism in mouse aortic SMC and found that gliotoxin caused cell detachment, whereas a negligible number of cells that remained attached were positive for cell death markers (propidium iodide) (Supplemental Figure 9). Gliotoxin treatment (1 μM) over 6 hours was associated with a 50% decrease in FAK phosphorylation without modification of total FAK protein level (Supplemental Figure 10). In the absence of gliotoxin (vehicle), shFAK induced morphological changes characterized by rounder cells but was insufficient to trigger cell detachment (Figure 5E). In contrast, shFAK markedly exacerbated gliotoxin-induced cell detachment compared with shScramble (shScr) (Figure 5, E and F). Beyond cell attachment, FAK KD exacerbated gliotoxin-induced apoptosis as evidenced by a higher percentage of Annexin V^+^ cells, increased Caspase-3 cleavage, and Caspase 3/7 activity (Figure 5, G–J, and Supplemental Figure 11). Notably, the acute downregulation of FAK did not reduce VCL and ILK protein levels (Supplemental Figure 10C), providing evidence that gliotoxin-induced anoikis exacerbation is directly attributable to FAK KD. However, we found that siRNA-mediated KD of VCL or ILK also increased gliotoxin-induced apoptosis (Figure 6). Notably, VCL KD was sufficient to induce significant cell death in the absence of gliotoxin, highlighting its critical role in SMC survival. Together, these experiments show that the expression level of focal adhesion proteins influenced SMC sensitivity to anoikis. FAK, ILK, and VCL downregulation, similar to that observed in human TAA, might promote SMC sensitivity to anoikis during aneurysm progression.

### FAK expression confers anoikis resistance independently of kinase function.

**Figure 7:**
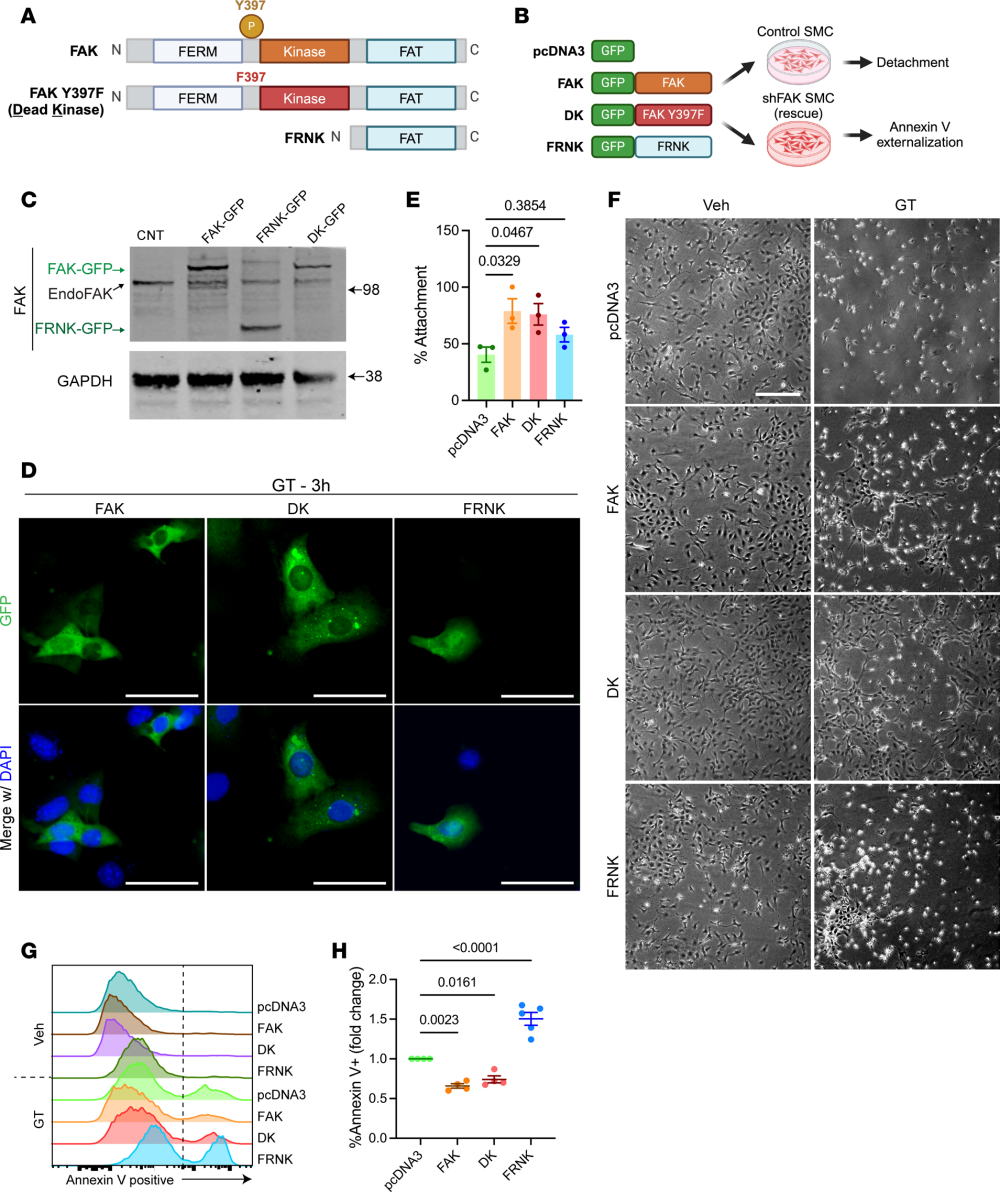
FAK confers protection against anoikis independently of kinase activity. (**A**) Schematic diagram showing the structure and functional domains of WT FAK, kinase-dead FAK (DK), and FAK-related nonkinase (FRNK). (**B**) Schematic of the experimental workflow for FAK overexpression. Control or shFAK-transduced SMCs are transfected with empty pcDNA3 vector or vectors encoding FAK, DK, or FRNK, followed by analysis of cell detachment or apoptosis. (**C**) Western blot showing overexpression of FAK, DK, and FRNK (fused with GFP) in SMC. (**D**) GFP signal in SMC transfected with FAK-GFP, DK-GFP, and FRNK-GFP revealing a cytoplasmic localization of the expressed constructs. DAPI used for nucleus staining. Scale bar: 50 μm. (**E**) Quantification of cell detachment in SMC overexpressing FAK, DK, or FRNK treated with gliotoxin. Data were represented as the percentage of attached cells after gliotoxin treatment relative to vehicle-treated cells. One-way ANOVA. (**F**) Representative images showing morphological changes in SMC overexpressing FAK, DK, or FRNK treated with vehicle or gliotoxin for 6 hours. Scale bar: 200 μm. (**G**) Annexin V assay assessing gliotoxin-induced anoikis in shFAK SMCs overexpressing FAK, DK, or FRNK. Representative flow cytometry histograms of Annexin V–Alexa Fluor 647 staining. (**H**) Quantification of Annexin V^+^ cells, presented as fold change in percentage relative to empty vector control. One-way ANOVA.

**Figure 8:**
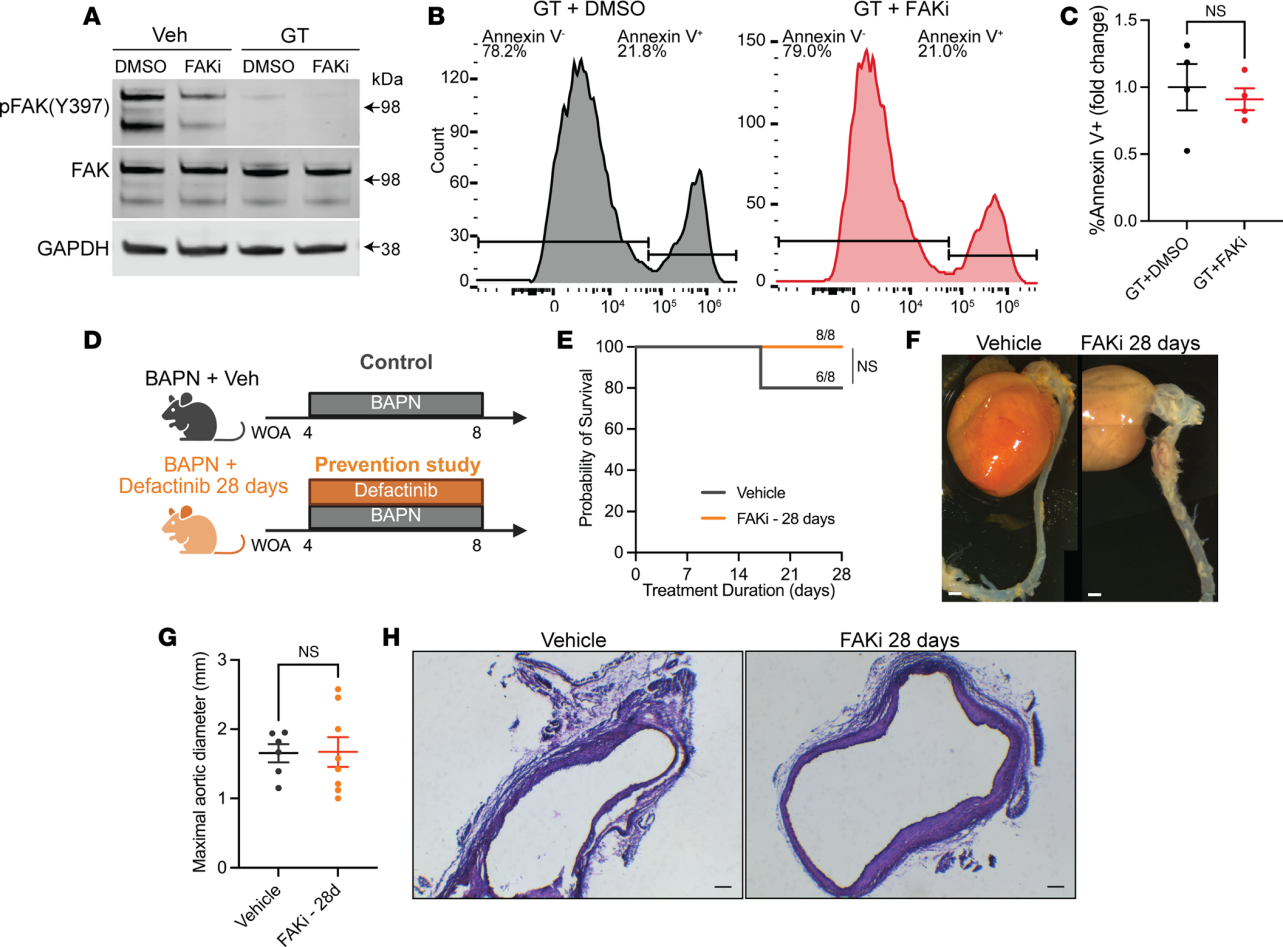
FAK kinase activity inhibition does not prevent gliotoxin-induced anoikis and aneurysm formation. (**A**) Representative images showing levels of phospho-FAK (Y397) and total FAK in mouse SMC pretreated with a FAK inhibitor (Defactinib: 1 μM, 24 hours) or DMSO, prior to incubation with Gliotoxin or Vehicle for 6 hours. GAPDH was used as a loading control (repeated 3 times, independently). (**B**) Representative flow cytometry histograms of Annexin V–Alexa Fluor 647 staining. (**C**) Quantification of Annexin V^+^ cells. Student’s *t* test. (**D**) Schematic diagram representing in vivo FAK inhibition studies. Twenty-eight-day-old mice were treated with BAPN and DMSO or Defactinib (20 mg/kg/day) for 28 days. (**E**) Survival probability was unchanged in mice treated with DMSO or FAKi. Kaplan-Meier test. (**F**) Representative images of the aortas of mice receiving FAKi or DMSO. Scale bar: 1 mm. (**G**) Measurement of the maximal aortic diameter (mm). Student’s *t* test. (**H**) H&E staining of aortic cross-sections. Scale bar: 100 μm.

We next aimed to investigate whether overexpressing or restoring expression of FAK protects SMC against anoikis. FAK is a multifunctional and multidomain protein that exerts its effects through kinase-mediated signaling (typically by phosphorylation on Y397) and through nonkinase structural functions (43). To dissect the role of these domains, residues, and posttranslational modification in anoikis resistance, WT FAK (FAK), dead-kinase FAK (DK), and FAK Related Non Kinase (FRNK) (Figure 7A) fused to GFP were expressed in control or shFAK SMCs as outlined in the experimental workflow in Figure 7B (44, 45). Western blotting confirmed the expression of the GFP-FAK constructs (Figure 7C and Supplemental Figure 12). FAK, DK, and FRNK constructs were mainly expressed in the cytoplasm, with no evidence of nuclear accumulation (Figure 7D). Importantly, we found that overexpression of either FAK or DK greatly reduced cell detachment after 6 hours of gliotoxin treatment (Figure 7, E and F). FRNK overexpression did not significantly change the percentage of adherent cells. Next, we tested whether FAK expression rescued shFAK-mediated SMC detachment and apoptosis. Annexin V flow cytometry analysis revealed that FAK and DK expression reduced the percentage of apoptotic shFAK SMCs after gliotoxin treatment by 30% (Figure 7, G and H). In contrast, cells expressing FRNK exhibited a marked increase (~1.5-fold) in Annexin V^+^ cells. These results demonstrate that FAK expression confers protection against anoikis, even in the absence of FAK Y397 phosphorylation. To further investigate the role of FAK kinase activity, we treated SMCs with Defactinib (1 μM), a potent FAK inhibitor (FAKi), thereby preventing FAK phosphorylation and activation (Figure 8A). SMC pretreatment with FAKi did not change the effects of gliotoxin on SMC apoptosis rate (Figure 8, B and C). Finally, we administered Defactinib (20 mg/kg/day) to 28-day-old C57BL/6 male mice for 28 days, concurrently with BAPN treatment (Figure 8D). Unlike in the SMC-specific *Ptk2* KO, FAKi did not alter survival probability (Figure 8E). None of the FAKi-treated mice died prior to cull at 28 days of treatment. Morphologically, FAKi did not exacerbate or prevent aortic dilation and remodeling (Figure 8, F–H). Together, these findings provide evidence that FAK protein expression displays a protective anti-anoikis effect through kinase-independent functions.

## Discussion

Our studies provide evidence that downregulation of critical focal adhesion complex components in human TAAD, including ILK, VCL, and FAK, contributes to the progression of aortic aneurysm and dissection. Specifically, we observed that: (a) the focal adhesion protein FAK, ILK, and VCL are downregulated at the transcript and protein levels in aortas of patients with TAA; (b) inducible SMC-specific KO of *Ilk*, *Vcl*, and *Ptk2* in mice treated with BAPN for 28 days exacerbates TAAD progression and results in poorer overall survival, increased aortic dilation, greater adverse medial remodeling, and SMC rarefaction due to cell death; (c) FAK-, ILK-, and VCL-deficient SMCs are more susceptible to anoikis in vitro, and *Ptk2^SMC–/–^* animals exhibit higher medial cell apoptosis; and (d) FAK overexpression attenuates SMC susceptibility to anoikis, independently of its kinase activity. These findings suggest that loss of structural focal adhesion protein accelerates TAAD progression by reducing resilience to cellular detachment, exacerbating SMC apoptosis and rarefaction, and compromising aortic wall integrity.

Pathological manifestations of TAAD are largely driven by SMC phenotypic and functional changes. These alterations are at least partly linked to the cell’s developmental origin. SMCs comprised in the thoracic aorta are derived from several distinct embryonic territories including the cardiac neural crest (CNC), the secondary heart fields (SHF), and the somite (46). In the ascending aorta, fate-mapping studies have revealed an overlap between CNC- and SHF-derived SMCs, which is thought to drive aortic dilation (47). For example, there is compelling evidence of regional and embryonic origin–specific SMC dysregulation of the TGF-β and angiotensin pathways in syndromic aneurysms (48, 49). This spatial distribution of CNC- and SHF-derived SMCs mirrors the pathological gradient observed in TAA, in which the outer medial region exhibits more severe pathological changes (46). Indeed, CNC-derived SMCs predominantly occupy the inner layers, while SHF-derived SMCs reside in the outer layers of the ascending aorta. Our study shows a marked reduction in FAK, ILK, and VCL expression in the inner two-thirds of the media selectively, interrogating about potential embryonic-origin differences. Conditional KO of *Ilk* in CNC-derived cells, driven by the *Wnt1* promoter, resulted in embryonic lethality. These mice exhibited severe cardiac developmental defects, including failure of CNC cells to differentiate into SMCs, leading to cells with rounded morphology and disorganized actin fibers (21). Similarly, noninducible *Tagln*-Cre mediated Ilk KO was associated with aorta enlargement and dilation during embryonic development (20). *Wnt1-*Cre–dependent *Ptk2* KO resulted in impaired cardiac outflow tract morphogenesis (50). These reports suggest that ILK and FAK are both essential for the differentiation of CNC-derived cells and SMCs. However, whether developmental origin influences SMC focal adhesion protein expression, FAK activation, and alterations in mechanotransduction during TAAD development and progression in adults remains to be further explored.

The phenotypic heterogeneity of aneurysmal SMCs has been characterized using scRNA-seq and other transcriptomic approaches, such as multiplexed error-robust fluorescence in situ hybridization (MERFISH) (40, 51, 52). In sporadic patients with TAA, scRNA-seq studies identified distinct SMC populations spanning a spectrum of phenotypes, including contractile SMCs, stressed SMCs, and fibromyocyte-like clusters, which coexpress contractile and ECM-remodeling markers, such as *SERPINE1* and *FN1* (8, 53). Additionally, proliferative clusters are distinguished by high cyclin expression and reduced contractile marker levels. Altogether, these phenotypic states contribute to different processes and stages of the disease. Our secondary analysis of scRNA-seq data from TAA patient aortas revealed that focal adhesion proteins FAK, ILK, and VCL are transcriptionally downregulated. In MFS mouse models, Ilk and Vcl expression is also reduced. Integration of these results with published studies reveals the complexity and heterogeneity of the focal adhesion pathway dysregulation. In vivo studies demonstrate that increased fibronectin/integrin signaling exacerbates aortic dilation in MFS SMCs and mice (15–17). Inhibiting integrin signaling with GLPG0187 — a broad-spectrum inhibitor of RGD-recognizing integrin heterodimers — reduced aortic dilation in MFS mice (17). Notably, no changes in p-FAK activation were reported in these studies, raising the question of how integrin targeting regulates downstream focal adhesion proteins. Interestingly, a recent study reported that α5β1 signaling and FAK expression are diminished in acute aortic dissection (AAD) in a small patient cohort based on histochemical analysis, accompanied by increased apoptosis and caspase-3 activation (54). Our 3 BAPN-treated SMC-specific focal adhesion gene KO mouse lines presented greater aortic dilation, loss of ECM structural integrity, SMC apoptosis, and rarefaction, resembling the pathological features observed in patients with TAAD (54). These findings highlight the complex and likely spatial-, temporal-, and context-dependent dysregulation of the focal adhesion complex in aneurysmal SMC. We found that *Ptk2^SMC–/–^* induced a marked loss of VCL and ILK expression in SMCs in animals treated with BAPN. These results highlight the interconnection between several components of the focal adhesion complex. We also observed remarkable similarities between the effects of *Ptk2*, *Ilk*, and *Vcl* KO. However, the precise effect of downregulating a given protein on the integrity of the focal adhesion complex and on other focal adhesion protein functions is difficult to assess, particularly in vivo over 4 weeks of treatment. A spatial and temporal delineation of the focal adhesion complex structure and activity would further inform on the mechanisms driving aortic dilation exacerbation.

It has been postulated that anoikis, while a physiological process essential for maintaining cellular homeostasis, participates in the pathogenesis of vascular and cardiac remodeling (37). In the context of TAAD, pericellular proteolysis and activation of thrombin and plasmin exacerbate SMC detachment and subsequent anoikis (34, 37). Accumulation of proteases and an increase in fibrinolytic activity have been associated with areas of cystic medial degeneration where SMC apoptosis and rarefaction occur, strongly suggesting the process of cell death mediated by loss of SMC adhesion to the ECM (33, 35, 36, 55). In non-SMC systems, disruption of integrin engagement with ECM proteins like fibronectin inhibits FAK phosphorylation at Tyr397, leading to the suppression of prosurvival pathways (AKT and ERK) and activation of proapoptotic cascades, including RhoA/ROCK/JNK signaling and caspase-3 (31, 42). Conversely, increased FAK overexpression enhances resistance to anoikis in fibroblasts (56). Our study reveals that the protective effect of maintenance of FAK expression against anoikis was independent of its kinase activity, as FAK and the dead-kinase mutant exhibited similar benefits against cell detachment and apoptosis, suggesting a predominant scaffolding role of FAK within the focal adhesion complex. Notably, overexpression of FRNK, a dominant-negative inhibitor of FAK, further exacerbated apoptosis in FAK KD SMC. This result may reflect the inhibitory effect of FRNK on residual FAK, as the KD does not fully ablate FAK expression. Mechanistically, FRNK comprises the focal adhesion targeting (FAT) domain, which enables localization to focal adhesion complexes but lacks the N-terminal FERM domain, which is essential for interaction with the plasma membrane, integrins, and actin-related proteins (57, 58). Meanwhile, FAK inhibition had no significant effect on SMC sensitivity to gliotoxin-induced anoikis. Our in vivo data found the FAKi had no protective or detrimental effect on aortic dilation. In an abdominal aortic aneurysm (AAA) mouse model, FAKi protected against aneurysm progression by inhibiting FAK-dependent proinflammatory pathways in macrophages (59). The discrepancy with our study likely reflects the differences between TAAD and AAA pathogenesis. It is also important to note that FAK inhibition is a systemic approach impacting multiple cell types. Future studies should elucidate the cell- and embryonic-origin-specific effects of FAK inhibition and FAK downregulation.

### Limitations of the study

We acknowledge several limitations in our study that highlight opportunities for future investigation. First, while we provide compelling evidence that reduced expression of VCL, ILK, and FAK exacerbates aortic dilation in mice, our current approach does not allow us to resolve the temporal or stage-specific contributions of these focal adhesion proteins to TAAD progression. The inducible KO strategy used here targets all medial SMC, preventing from interrogating the heterogeneity of SMC subpopulations or assessing whether focal adhesion proteins exert distinct effects embryonic origins, phenotypic states, or disease stages. Cluster-specific or region-specific inducible Cre models would offer significant mechanistic insight but are not yet broadly available. Second, although the BAPN model successfully recapitulates key features of human TAAD, including ECM disorganization and SMC loss, it may not capture the full spectrum of TAAD pathophysiology. Additional validation of our findings in other experimental and genetic TAAD models will be necessary to extend our conclusions across contexts and etiologies. Lastly, we were unable to address sex as a biological variable due to the Y chromosome insertion of the *Myh11*-CreER^T2^ transgene used in our mouse model (60), which restricted our studies to male animals. This is a notable limitation, given the increasing evidence of sex-specific susceptibility to aneurysm formation in the BAPN model and human TAAD. Future studies should employ newly developed Cre systems allowing for SMC and vascular SMC-specific genetic targeting in both males and females (61, 62).

## Methods

### Sex as a biological variable.

This study used human aortic specimens from both male and female individuals. Data from these specimens have not been analyzed to statistically address sex as a biological variable. In mouse studies, experiments were conducted exclusively on male mice because the *Myh11*-CreER^T2^ transgene (Jackson Laboratory #019079) is located on the Y chromosome, making it impossible to use females (63).

### Human aortic specimens.

Human ascending aortic tissues were collected from deceased donors or from patients undergoing ascending aorta or aortic valve replacement. Aneurysmal [aortic diameter = 54 mm ± 6.047 (mean ± SD)] and nonaneurysmal (aortic diameter ≤ 44 mm) ascending aortas were collected from age-matched males and females (Supplemental Figure 2). Aortic specimens were dissected and fixed overnight in a 4% paraformaldehyde solution before processing, paraffin-embedding, and sectioning (5 μm-thick cross-sections).

### Mice.

Mice were housed in routinely sanitized cages under controlled temperature (20°C–26°C) and humidity (50%–60%) with a 12-hour light/dark cycle and ad libitum access to standard rodent chow and water. *Myh11*-CreER^T2^ (Jackson Laboratory #019079) and R26R-YFP (Jackson Laboratory #006148) mouse lines were crossed to obtain *Myh11*-CreER^T2^-YFP lineage tracing mice as previously described (60). *Myh11*-CreER^T2^-YFP;*Ptk2^flox^*, *Myh11*-CreER^T2^-YFP;*Ilk^flox^*, and *Myh11*-CreER^T2^-YFP;*Vcl^flox^* mice were generated by crossing *Myh11*-CreER^T2^-YFP mice with *Ptk2^flox^* (Jackson Laboratory #031956), *Ilk^flox^* (Jackson Laboratory #023310), and *Vcl^flox^* (Jackson Laboratory #028451). All strains were on a C57BL/6J background, and mice were genotyped by PCR: Ptk2-45213: GAACTTGACAGGGCTGGTCT; Ptk2-45214: CTCCAGTCGTTATGGGAAATCT; Ilk-17598: GACCAGGTGGCAGAGGTAAG; Ilk-17599: GCTTTGTCCACAGGCATCTC; Vcl-26403: CATCATGAGTTCTTGACCTGGA; Vcl-26404: TGCAAACCCTAATAATTTTACGAAC. Male mice were exclusively used as experimental animals due to the location of the Myh11-CreERT2 transgene on the Y chromosome. Breeding between wt/flox heterozygous mice allowed for the generation of wt/wt, wt/flox, and flox/flox experimental littermates.

Three to 4-week-old mice were simultaneously treated with tamoxifen and BAPN monofumarate (Sigma Aldrich #A3134). Mice received 10 daily injections of 1 mg tamoxifen in peanut oil (10 mg/mL). BAPN (0.4%, 1g/kg/day) was delivered in drinking water and refreshed weekly to induce aortic dilation, as previously described (64). Mice were euthanized, and aortas were collected after 28 days of BAPN treatment. In brief, the aorta was isolated, and the ascending aorta was dissected and incubated in 4% paraformaldehyde overnight before processing, paraffin embedding, and sectioning.

### Cell culture.

A primary mouse aortic SMC line was generated by our lab from C57BL/6 mice and was cultured in Dulbecco’s Modified Eagle Medium (DMEM) supplemented with 10% fetal bovine serum (FBS), 1% penicillin-streptomycin, and 1.6 mM L-glutamine at 37°C in a humidified atmosphere with 5% CO_2_. Before stimulation and for baseline measurements, SMC were starved in serum-free, insulin-free medium supplemented with 1.6 mM L-glutamine, 0.2 mM L-ascorbic acid (Sigma Aldrich, A4403), 5 μg/mL Apo-Transferrin (Sigma Aldrich, T5391), and 6.25 ng/mL Na Selenite (Sigma Aldrich, S5261) for 24 to 48 hours. SMC from 3–5 passages were used for independent experiments. Cells were treated with 1 μM gliotoxin (Sigma, G9893) for up to 6 hours to induce anoikis.

### Cell transduction and transfection.

For FAK KD, SMCs were transduced with lentiviral vectors pLV-shRNA-mCherry:T2.FAK.1 (VectorBuilder, VB191112-1506sar) or control pLV-shRNA-mCherry:T2A.Scramble (VectorBuilder, VB191112-1483abp). Lentivirus was packaged using a third-generation system and titrated using the Lenti-X qPCR Titration Kit (Takara) following the manufacturer’s protocol. Cells were transduced at 10^6^ vg/mL using Polybrene (Sigma, TR-1003-G) and selected with 5 μg/mL Puromycin. Transient overexpression of FAK-GFP (Addgene #50515), FAK-Y397F-GFP (Addgene #50516), FRNK-GFP (Addgene #50518), or pCDNA3-GFP control (Addgene #74165) was performed using Fugene HD and following the manufacturer’s protocol (Promega, E2311).

For VCL and ILK silencing, 70% confluent mouse aortic SMCs were transfected with siILK (ThermoFisher, siRNA, 159172), siVCL (ThermoFisher, siRNA, 65290), or the negative control (ThermoFisher, AM4611) using Lipofectamine RNAiMax Reagent (ThermoFisher, 13778150) according to the manufacturer’s instructions. Cells were cultured in medium containing 10% FBS for 24 hours after transfection and were then treated with gliotoxin (1 μM) for 6 hours.

### Bioinformatic analysis.

scRNA-seq datasets were acquired from the gene expression omnibus (GEO) repositories GSE155468 (human thoracic aortic aneurysm specimens) and GSSE153534 (murine MFS aortic aneurysm). The preprocessed gene expression matrices) were imported into the Seurat package in R. Genes detected > 3 cells and cells with > 200 distinct genes were included in the analysis. Doublets, clumps, and free RNA were excluded from the analysis based on cell read counts and genes. Cells with > 7.5% mitochondrial gene content were excluded. Data normalization, scaling, and regression were performed by SCTransform in Seurat package. Principle component analysis and nonlinear dimensional reduction using uniform manifold approximation (UMAP) and t-distributed stochastic neighbor embedding (t-SNE) were performed to reduce the data dimension. Cell cluster gene expression signatures were analyzed by FindAllMarkers function in Seurat via nonparametric Wilcoxon rank-sum test using default settings. Pathway analysis of differentially expressed genes between SMC and modSMC by ClusterProfiler using default parameters.

### IHC.

Human aortic sections were deparaffinized and subjected to antigen retrieval (Vector Laboratories, H-3300). Sections were blocked with BLOXALL Blocking Solution (Vector Labs) for 10 minutes, followed by a 30-minute incubation in 2.5% Normal Goat Serum. Primary antibodies FAK (Millipore, #05-537, clone 4.47, mouse monoclonal), ILK (Abcam, #ab76468, clone EPR1592, rabbit monoclonal), and VCL (Abcam, #ab129002, clone EPR8185, rabbit monoclonal) were applied overnight at a 1:200 dilution at 4°C. Sections were then incubated with ImmPRESS Polymer Reagent (Vector Labs) for 30 minutes, followed by DAB staining using ImmPACT DAB EqV (Vector Labs) until the desired intensity was achieved. Finally, sections were dehydrated, cleared, and mounted with xylene-based mounting media. Image analysis was conducted using ImageJ and Image Pro Premier software.

Mouse aortic sections were stained using the Verhoeff-Van Gieson (VVG) method (Polysciences, 25089-1). Sections were first incubated in Verhoeff’s solution for 15 minutes. Sections were rinsed in tap water with 3 changes, followed by differentiation in 2% ferric chloride for 4 minutes. Sections were then washed in tap water for 5 minutes and incubated with 5% sodium thiosulphate for 1 minute. Sections were washed under running tap water for 5 minutes, followed by counterstaining with Counterstain I solution for 3–5 minutes. Dehydration was performed using 95% ethanol for 5 minutes, followed by 2 changes of 100% ethanol, and cleared in 2 changes of xylene, each for 3 minutes. Slides were then mounted with mounting medium (VectaMount Express, H-5700-60). VVG-stained images were captured using a PreciPoint scanner. Mouse aortic sections were also stained using the H&E method as previously described (65). Image analysis was conducted using ImageJ and Image Pro Premier software.

### Aortic dilation analysis.

High-resolution ultrasound imaging was performed to capture longitudinal and cross-sectional images of the ascending aorta, aortic arch, and abdominal aorta using the Vevo 3100 ultrasound system (VisualSonics Inc.). Additional ascending and descending aortic diameter measurements were taken during tissue collection.

### Immunofluorescence.

Mouse aorta cross-sections (10 μm thickness) were deparaffinized and subjected to antigen retrieval. Sections were blocked with 10% horse serum and 0.6% Fish Gelatin (FSG) in PBS for 1 hour. Sections were incubated with primary antibodies against GFP (Santa Cruz, #sc-9996, clone B-2, mouse monoclonal), ACTA2-FITC (Sigma, #F3777, clone 1A4, mouse monoclonal), ILK (Abcam, #ab76468, clone EPR1592, rabbit monoclonal), or VCL (Abcam, #ab129002, clone EPR8185, rabbit monoclonal), followed by donkey anti-mouse 555 secondary antibodies (Invitrogen, #A31570, donkey polyclonal). Nuclei were counterstained with DAPI.

TUNEL staining was performed following the manufacturer’s protocol (Invitrogen, C10619). Briefly, after deparaffinization, tissue sections were permeabilized by Proteinase K solution for 15 minutes at room temperature. Terminal deoxynucleotidyl transferase (TdT) reaction was performed by adding a prepared TdT reaction mixture, including EdUTP and TdT enzyme, to each slide and incubating for 60 minutes at 37°C. Immunofluorescent labeling of EdUTP was performed by adding Alexa Fluor 647 picolyl azide dye and copper protectant and incubating for 30 minutes at 37°C in the dark. Nuclei were counterstained with DAPI (1: 500).

Slides were mounted with Prolong Gold Antifade Reagent (Fisher, P36930) and visualized using a Leica DMi8 fluorescence or Nikon A1 confocal microscope. Image analysis was conducted using ImageJ and Image Pro Premier software.

### qPCR.

Total RNA was extracted from cells using Trizol reagent (Invitrogen, 15596026), according to the manufacturer’s instructions. RNA concentration and purity were assessed using the Qubit RNA Broad Range Assay Kit (Invitrogen, Q10210). cDNA synthesis was performed using the iScript cDNA Synthesis Kit (Bio-Rad, 1708891). qPCR was carried out using the SYBR Green PCR system (Applied Biosystems, A25742). *Ptk2* transcript levels were normalized to Gapdh using the ΔΔCT method. Primer sequences used for qPCR are: mPtk2-F ACACTTGGAGAGCTGAGGTC, mPtk2-R GACACCAGAACATTCCGAGC, mGapdh F GTTGTCTCCTGCGACTTCA, mGapdh R GGTGGTCCAGGGTTTCTTA.

### Immunoblotting.

Proteins were extracted from cells using RIPA lysis buffer (Sigma, 11836170001) containing protease and phosphatase inhibitors (Sigma, P5726-1ML, P0044-1ML, 1:1,000). Lysates were heated in reducing LDS sample buffer (Invitrogen, NP0007) at 95°C for 10 minutes. Equal amounts of protein were separated by SDS-PAGE on 4–12% Bis-Tris gels (Invitrogen, NP0321BOX) and transferred onto 0.45 μm nitrocellulose membranes (Bio-Rad, 1620115). Membranes were incubated with primary antibodies (dilution 1:1,000 if not explicitly mentioned otherwise) against phospho-FAK (Tyr397) (Thermo Fisher, #44-624G, rabbit polyclonal), FAK (Cell Signaling, #3285S, rabbit polyclonal), ILK (Abcam, #ab76468, clone EPR1592, rabbit monoclonal), VCL (Abcam, #ab129002, clone EPR8185, rabbit monoclonal), cleaved-caspase 3 (Cell Signaling, #9661, Rabbit polyclonal), caspase-3 (Cell Signaling, #14220, clone D3R6Y, rabbit monoclonal), and GAPDH (Santa Cruz, #sc-365062, clone G-9, mouse monoclonal, dilution: 1:5000), followed by incubation with IRDye secondary antibodies (LI-COR). Visualization was performed using the LI-COR Odyssey CLx imaging system.

### Proliferation assays.

SMC proliferation was assessed using a BrdU incorporation assay. Cells were incubated with BrdU (10 μg/mL) for 6–16 hours, followed by fixation in 4% paraformaldehyde (PFA) and permeabilization with 0.2% Triton-X-100 (Sigma). DNA was denatured with 2N HCl (Sigma, 143007) for 30 minutes. Cells were incubated with anti-BrdU antibodies (Abcam, ab6326) and stained with secondary donkey anti-rat 555 antibodies (Abcam, ab150154).

Cell cycle analysis was also performed using flow cytometry. VSMCs were harvested using trypsin and fixed in 75% cold ethanol. Following the manufacturer’s instructions, cells were stained with the Propidium Iodide Flow Cytometry Kit (Abcam, ab139418). All samples were analyzed using a Cytek Aurora System. Data analysis was performed using FlowJo v10.10 software.

### Apoptosis assays.

Gliotoxin-treated SMCs were imaged (bright-field microscopy) at different time points. Detachment was assessed by measuring the cell-free area using ImageJ (NIH).

Both adherent and floating cells were collected after gliotoxin treatment for apoptosis detection by flow cytometry. In total, 1 × 10^6^ cells in suspension were stained with Annexin V–Alexa Fluor 647 (Invitrogen, A23204, 1:50) and DAPI (Invitrogen, D3571, 0.01 mg/mL) and incubated for 15 minutes at room temperature in the dark before data collection by flow cytometry as mentioned in the proliferation assay protocol. Data were analyzed in FlowJo v10.10 software. For gating, forward scatter (FSC) versus side scatter (SSC) was used to set an initial gate on live, single cells to exclude debris aggregates outside the normal FSC/SSC range. Further doublet exclusion was performed by using FSC-A versus FSC-H plot.

Finally, apoptosis was measured using the Caspase-Glo 3/7 Assay System (Promega, G8091) based on the manufacturer’s protocol. Briefly, following gliotoxin treatment, 2 aliquots of 1 × 10^4^ to 5 × 10^4^ cells were collected after trypsinization. One aliquot was mixed with an equal volume (1:1) of Caspase-Glo 3/7 reagent and incubated for 30 minutes at room temperature. Luminescence was measured using a BioTek Synergy H1 plate reader. Protein concentration was determined on the second aliquot by BCA assay. Luminescence values were normalized to protein concentration.

### FAK inhibition studies.

In vivo FAK inhibition was performed by treatment with the FAK inhibitor Defactinib as previously described (66). Four-week-old C57BL/6J mice (Jackson Laboratory, #000664) were treated with BAPN (0.4%, ~1 g/kg/day) delivered in drinking water. Defactinib (VS-6063; MedChemExpress) was dissolved in 10%DMSO in corn oil and administered daily by oral gavage (20 mg/kg/day). Defactinib treatment was initiated at the same time than the BAPN treatment and continued for 28 days.

In vitro, after reaching 60%–70% confluence, SMCs were washed with PBS and incubated in serum-free medium containing 1 μM Defactinib (or DMSO as a vehicle control) for 24 hours. Subsequently, SMCs were harvested for protein analysis or treated with Gliotoxin (1 μM, 6 hours) for Annexin V apoptosis assay as previously described.

### Statistics.

Data are presented as the mean ± SEM. In vitro experiments were repeated independently at least 3 times with duplicated technical repeats. One data symbol represents the mean value of technical repeats for 1 experiment. For in vivo experiments, mouse littermates were used for each group. The number of mice is provided in individual figure legends. All statistics were performed using GraphPad Prism 10. Two-tailed unpaired Student’s *t* tests with 95% CI were used to compare 2 groups with continuous variables with normal distribution and equal variances tested by the Kolmogorov-Smirnov test. Two-tailed unpaired Student’s *t* tests followed by a Welch’s correction with a confidence level of 95% were performed if 2 groups had unequal variances. Two-tailed unpaired Mann-Whitney *U* tests with a confidence level of 95% were used if variables were nonnormally distributed. For comparison between multiple groups with a single factor or 2 factors, we used 1-way or 2-way ANOVA, respectively. The probability of survival was analyzed using Kaplan-Meier. Wilcoxon test was used to compare gene expression derived for scRNA-seq. *P* < 0.05 was considered as statistically significant.

### Study approval.

Human studies were approved by the University of Pittsburgh IRB in accordance with the Declaration of Helsinki. Animal experiments were approved by the IACUC at the University of Pittsburgh and conducted in an American Association for Accreditation of Laboratory Animal Care–accredited facility.

### Data availability.

Values for all data points in graphs are reported in the Supporting Data Values file. The data that support the findings of this study are available from the corresponding author upon reasonable request.

## Author contributions

DG and CED conceived the project. DG, CED, ZZ, RE, and CSH designed the experiments. ZZ, CED, ML, JW, DS, PB, and RE performed experiments and analyzed the data. JAP and CSH provided human specimens. DG, CED, and ZZ wrote the manuscript. All coauthors edited the final version of the manuscript.

## Conflict of interest

The authors have declared that no conflict of interest exists.

## Funding support

This work is the result of NIH funding, in whole or in part, and is subject to the NIH Public Access Policy. Through acceptance of this federal funding, the NIH has been given a right to make the work publicly available in PubMed Central.

- NIH R01HL146465 and R01HL166425 (to DG).
- American Heart Association 20IPA35310394 grants (to DG).
- NIH T32 HL 129964-6 A1 (to CED).
- American Heart Association 23CDA1044815 (to CED).
- National Institute of Health grants R56HL168657 and R01HL176595 (to CSH).
- American Heart Association 24POST1186619 (to PB).
- McKamish Family Foundation (to CSH).

## Supplementary Material

Supplemental data

Unedited blot and gel images

Supporting data values

## Version 1 03/24/2026

In-Press Preview

## Version 2 05/08/2026

Electronic publication
