# Supplementary material for: Focal adhesion proteins confer smooth muscle anoikis resistance and protection against aortic aneurysm and dissection
Source: JCI Insight. 2026 Mar 24;11(9):e195291. doi: 10.1172/jci.insight.195291 (PMC13232016; doi:10.1172/jci.insight.195291)
Supplement: Supplemental data [file jciinsight-11-195291-s023.pdf]

Supplementary Figures

Focal Adhesion proteins confer smooth muscle anoikis resistance and protection against aortic aneurysm and dissection

Zhenyuan Zhu, Mingjun Liu, Jianxin Wei, Deepa Suryanarayan, Parya Behzadi, Robert Edgar, Julie Phillippi, Cynthia St. Hilaire, Cristina Espinosa Diez, Delphine Gomez

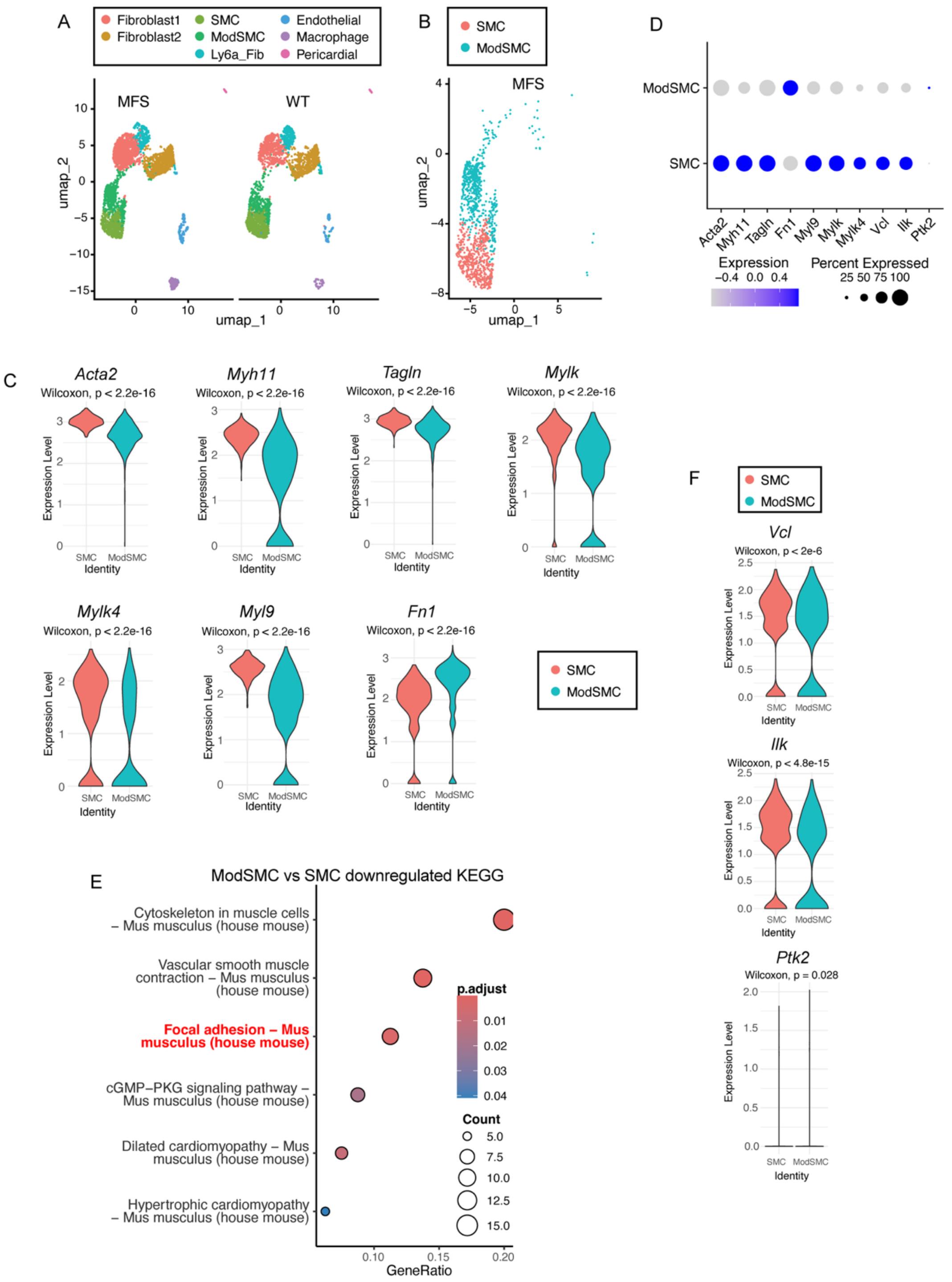

<u>Supplementary Figure 1:</u> Vinculin and ILK transcripts are downregulated in modulated aortic SMC from *Fbn1*$^{+/C1041G}$ mice. A. Uniform Manifold Approximation and Projection (UMAP) dimensional reduction plot of all aortic cell populations derived from Marfan Syndrome (MFS) mice (n=4) and Wild Type (WT) mice (n=3) based on data from GSE153534. **B.** UMAP representation displaying smooth muscle cells (SMC) and modulated smooth muscle cells (ModSMC) subclusters in the aorta of MFS

mice. **C.** Violin plots representing *Acta2*, *Myh11*, *Tagln*, *Mylk*, *Mylk4*, *Myl9*, and *Fn1* transcript expression levels in SMC and modSMC subclusters. Wilcoxon test. **D.** Dot plot illustrating the expression of contractile- (Acta2, Myh11, Mylk), synthetic- (Fn1) and focal adhesion-associated transcripts (Vcl, Ilk) in SMCs compared to ModSMCs. **E.** KEGG pathway analysis depicting the most significantly downregulated pathways in ModSMCs versus SMCs in MFS. **F.** Violin plot depicting *Vcl*, *Ilk*, and *Ptk2* transcript levels in SMC and ModSMC subclusters. Wilcoxon test.

		Healthy donors	TAA	Statistics
**Number**	Total	n=8	n=8	n.s.
	Male	n=4	n=4	n.s.
	Female	n=4	n=4	n.s.
**Age (years)**	Average	45.38	45.5	n.s.
	SD	5.63	8.864	
**Aortic diameter (mm)**	Average	-	54	
	SD	-	6.047	

Supplementary Figure 2: **Healthy and TAA aortic sample cohort.** The numbers, sexes, and ages of the human specimens used for histological analysis are presented in Figures 1 and 3. The diameter of healthy aortas was not available. However, these samples did not present aortic dilation, enlargement, or tortuosity.

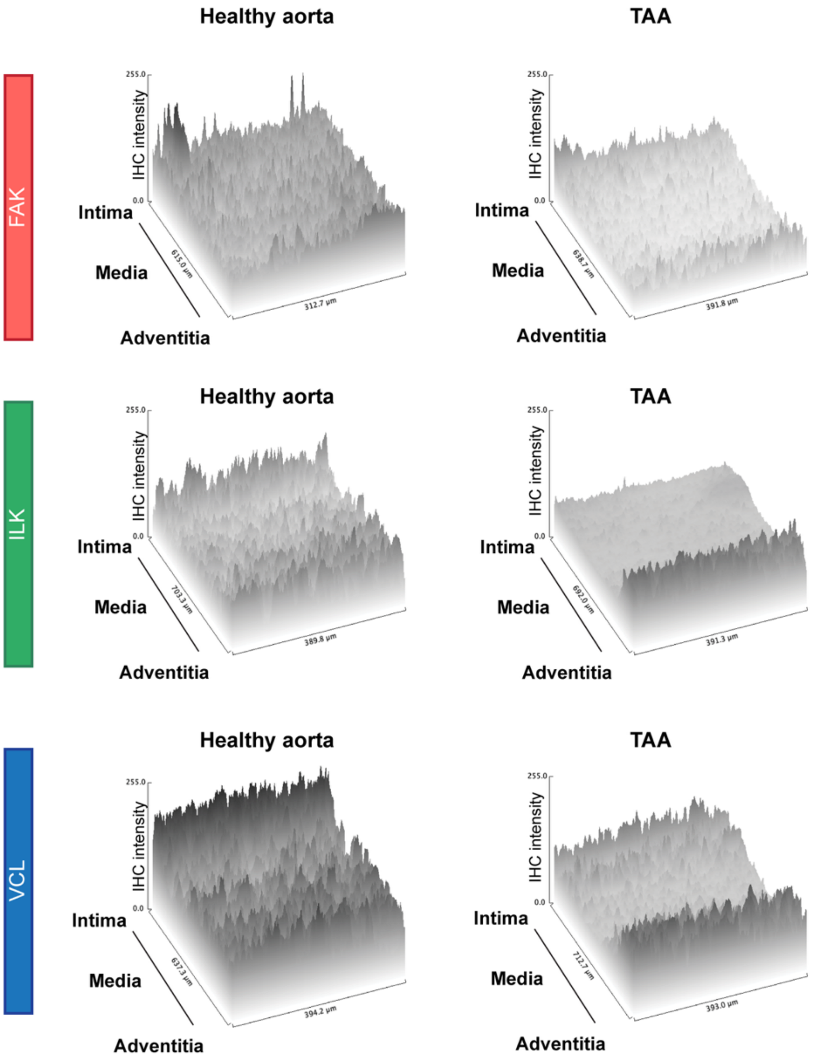

<u>Supplementary Figure 3:</u> FAK, ILK, and VCL are downregulated and heterogeneously expressed in human TAA aortas. FAK, ILK, and VCL IHC staining intensity plots across the vessel wall thickness in healthy control and TAA aortas (y axis units: IOD; x and z axis: μm).

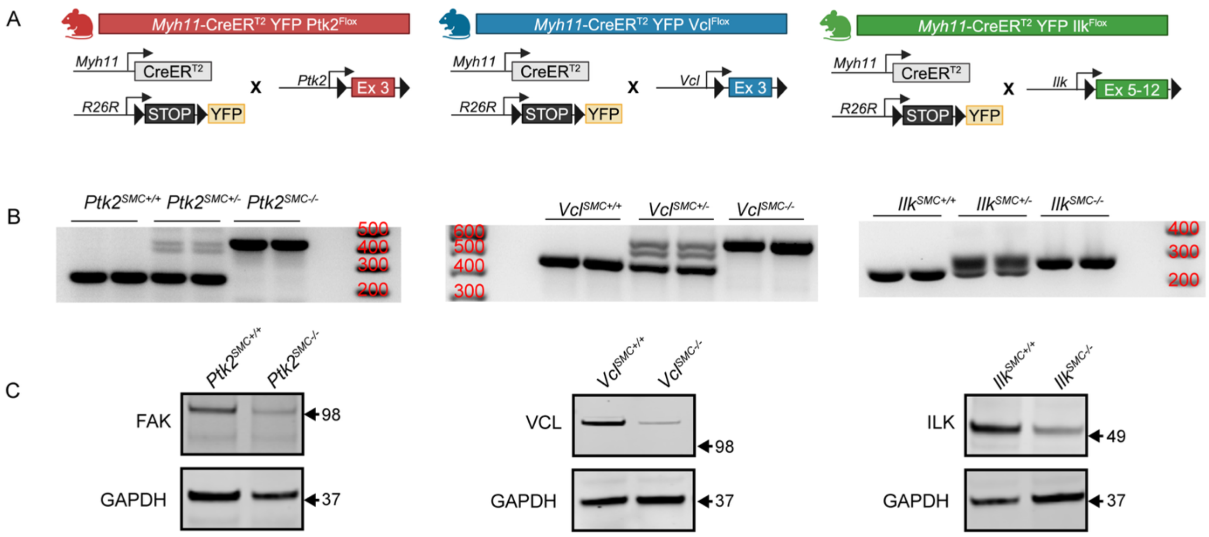

<u>Supplementary Figure 4:</u> Generation and validation of SMC-specific Focal Adhesion protein knockout mice. A. Breeding scheme illustrating the generation of SMC-specific *Ptk2, Vcl*, and *Ilk* KO mice. *Ptk2*^{Flox} (Jackson Laboratory #031956), *Vcl*^{Flox} (Jackson Laboratory #028451), and *Ilk*^{Flox} (Jackson Laboratory #023310) mice were crossed with *Myh11*-CreER^{T2} YFP mice (Jackson Lab #019079 and #006148). B. Representative images of SMC-specific *Ptk2*, *Vcl*, and *Ilk* KO mouse line genotyping. With a 100 base pair DNA ladder as a size reference. All mice used in the study were genotyped at weaning. C. Representative Western blot of VCL, ILK, and FAK in *Ptk2*$^{SMC-/-}$, *Vcl*$^{SMC-/-}$, and *Ilk*$^{SMC-/-}$ mice. GAPDH was used as housekeeping protein. Western blot analyses have been conducted on n=4 per genotype.

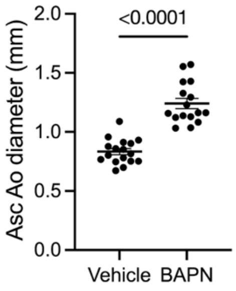

<u>Supplementary Figure 5:</u> BAPN treatment induces aortic dilation. Maximal diameter of the ascending aorta in 28-day-old mice treated with BAPN or vehicle for 28 days.

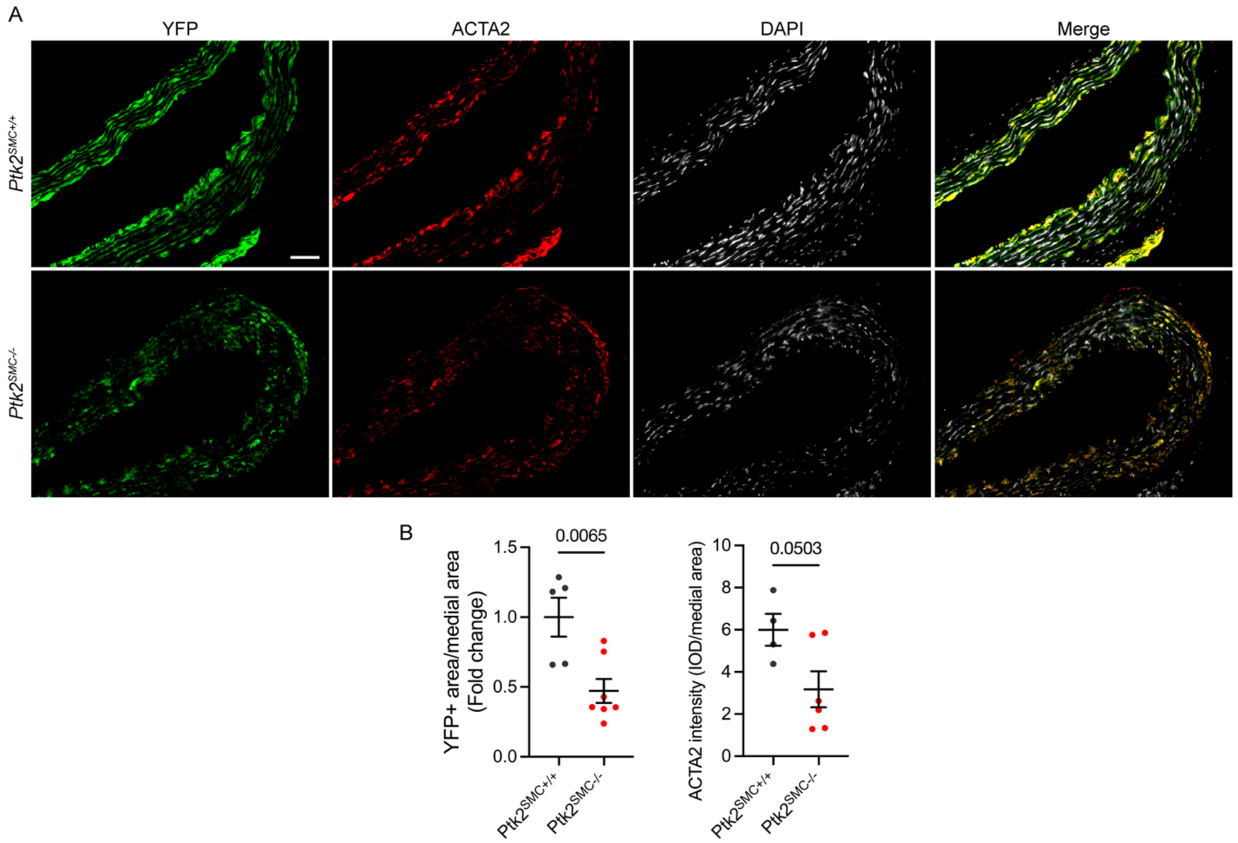

<u>Supplementary Figure 6:</u> A. Representative micrographs of YFP (green), ACTA2 (red) and DAPI (white) staining in aortic cross-sections from *Ptk2*$^{SMC+/+}$ and *Ptk2*$^{SMC-/-}$. Scale bar: 100 μm. **B.** Quantification of YFP+ medial area and ACTA2 intensity (integrated optical density). Student t-tests.

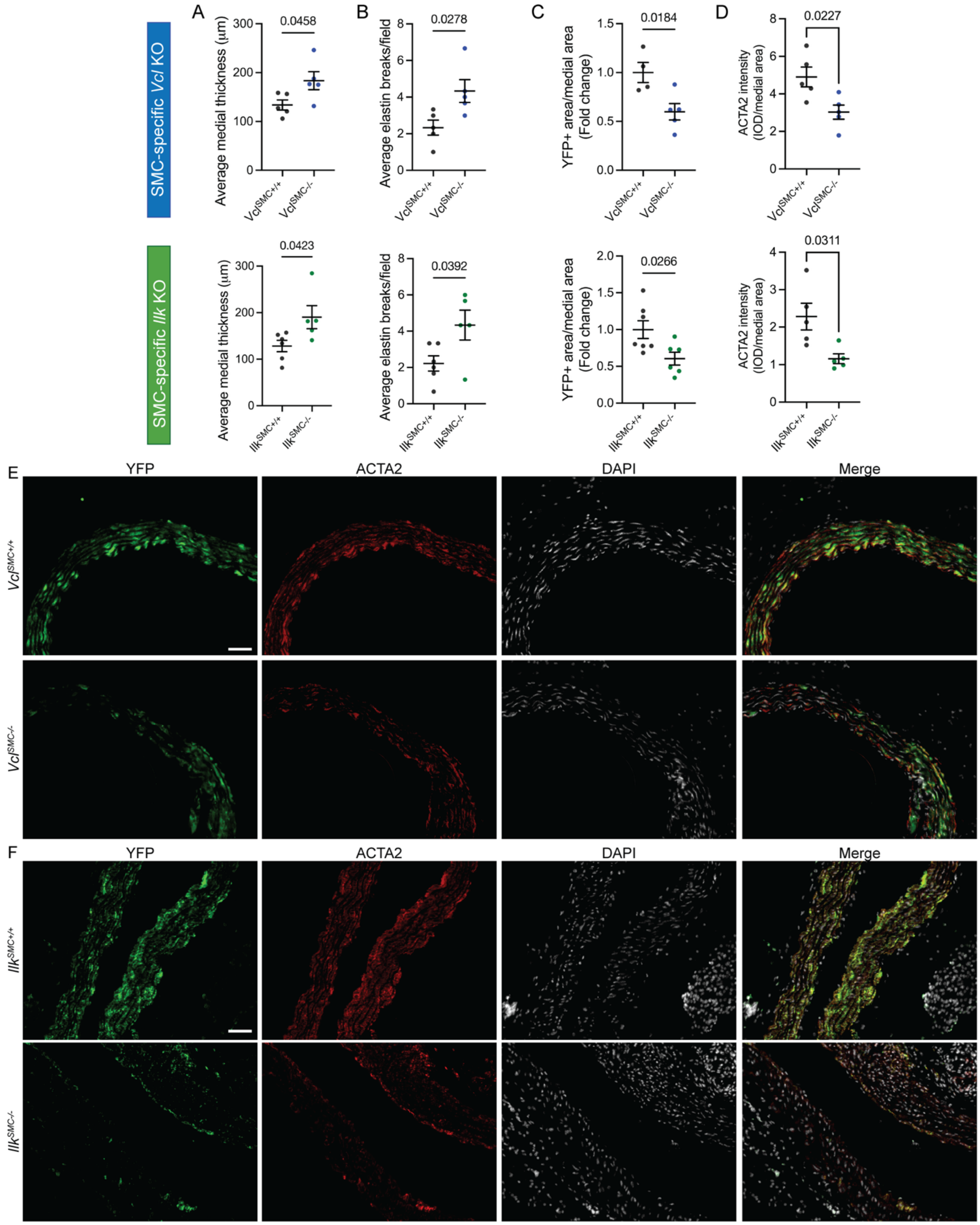

Supplementary Figure 7: Deletion of VCL and ILK in SMC exacerbates detrimental vessel and extracellular matrix remodeling induced by BAPN. A. Medial thickness in the ascending aorta of *Vcl*$^{SMC-/-}$ and *Ilk*$^{SMC-/-}$ mice compared to their respective WT littermates. Student t-test. **B.** Quantification of

elastin breaks. Student t-test. **C.** Quantification of YFP+ medial area. Student t-tests. **D.** Quantification of ACT2 IF staining (integrated optical density) normalized to medial area. Student t-test. **E.** Representative micrographs of YFP (green), ACTA2 (red), and DAPI (white) staining in aortic cross-sections from $\mathit{Vcl}^{SMC+/+}$ and $\mathit{Vcl}^{SMC-/-}$ mice (scale bar = 100 μm). **F.** Representative micrographs of YFP (green), ACTA2 (red), and DAPI (white) staining in aortic cross-sections from $\mathit{Ilk}^{SMC+/+}$ and $\mathit{Ilk}^{SMC-/-}$ mice (scale bar = 100 μm).

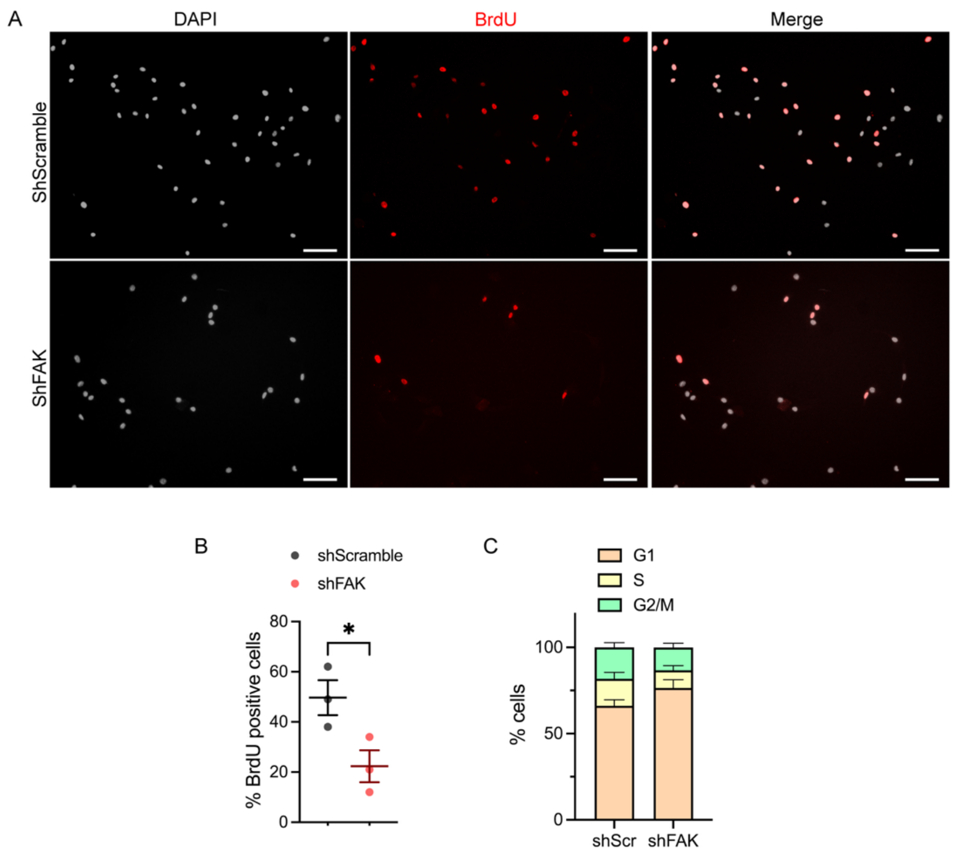

<u>Supplementary Figure 8:</u> FAK knockdown decreases SMC proliferation. A. Representative images of BrdU staining. Nuclei are stained with DAPI. Scale bar: 50 μm. **B.** Percentage of BrdU incorporation in ShScramble and ShFAK SMC. Student's t-test. * $p < 0.05$. **C.** Cell cycle analysis by flow cytometry determined the proportion of cells in G1, S, or G2/M phase.

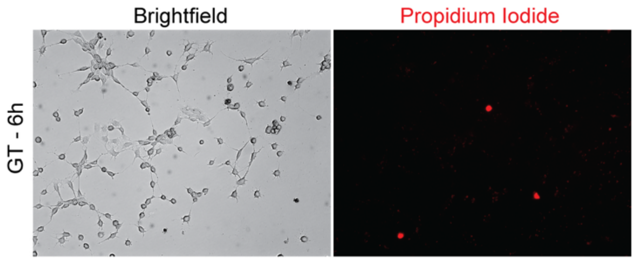

<u>Supplementary Figure 9:</u> Gliotoxin treatment induces cell detachment. Brightfield and Propidium Iodine (PI) staining in mouse SMC treated with Gliotoxin (1 μM) for 6 hours. Gliotoxin induced cell detachment. Cells that did not detach were largely negative for PI, a cell death marker.

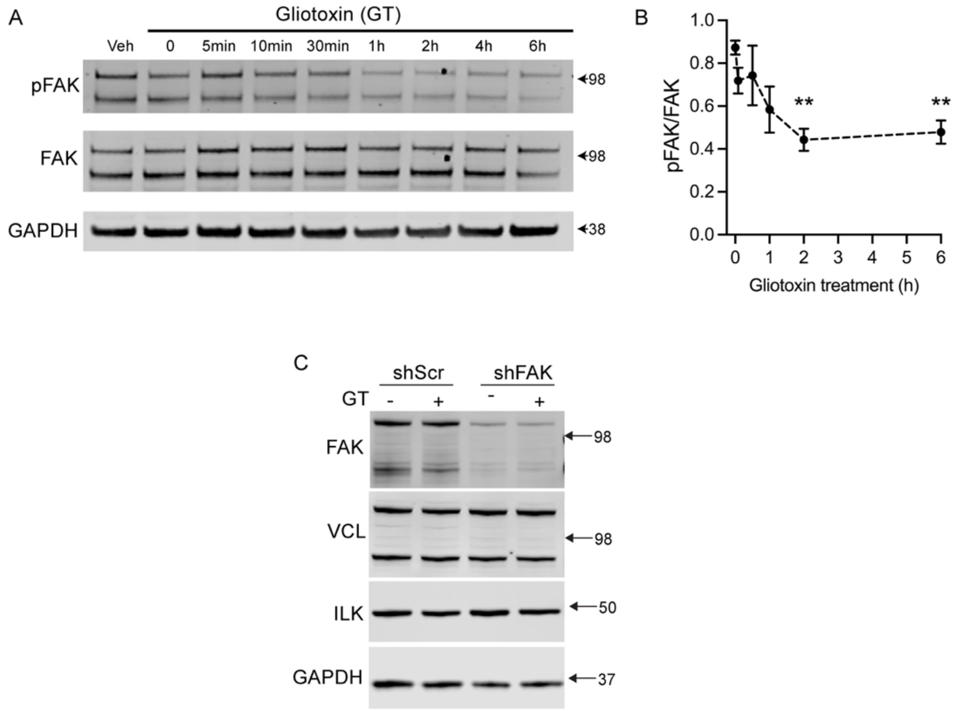

<u>Supplementary Figure 10:</u> **Gliotoxin-induced anoikis is associated with decreased FAK phosphorylation. A.** Representative Western blot showing phosphorylation level of FAK on Y397 in control SMCs (lane 1) and SMCs treated with Gliotoxin (1 μM) over 6 hours. GAPDH was used as a loading control. Western Blot analyses have been conducted on 3 independent experiments. **B.** Densitometric analysis of phosphorylation level of FAK from three independent experiments. Data were shown as the ratio of phosphorylated FAK to total FAK. One-way ANOVA. ** $p < 0.001$ compared to baseline. **C.** Representative Western Blot representing the protein expression level of FAK, VCL, and ILK after FAK silencing (shFAK) and treatment with Gliotoxin (1 μM) for 6 hours. GAPDH was used as a loading control. Western Blot analyses have been conducted on 3 independent experiments.

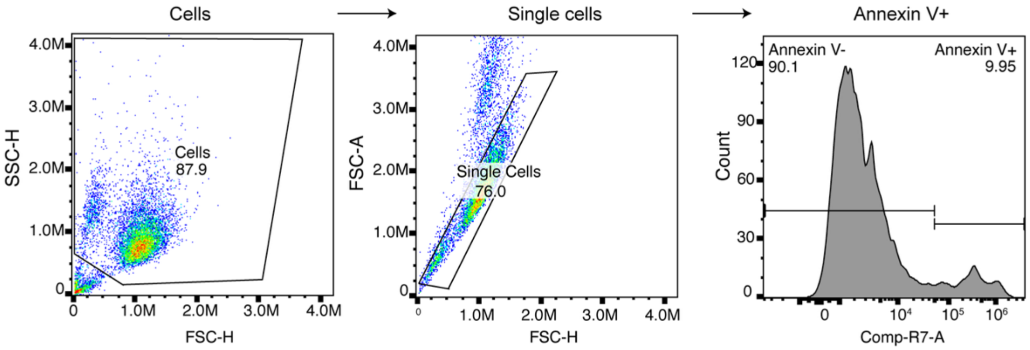

<u>Supplementary Figure 11:</u> Annexin V flow cytometry analytical pipeline. Cell gating for Annexin V$^+$ cell quantification. Debris and cell doublets are excluded prior to quantification of Annexin V$^+$ and Annexin V$^-$ SMC. This pipeline was used for all flow cytometry analyses.

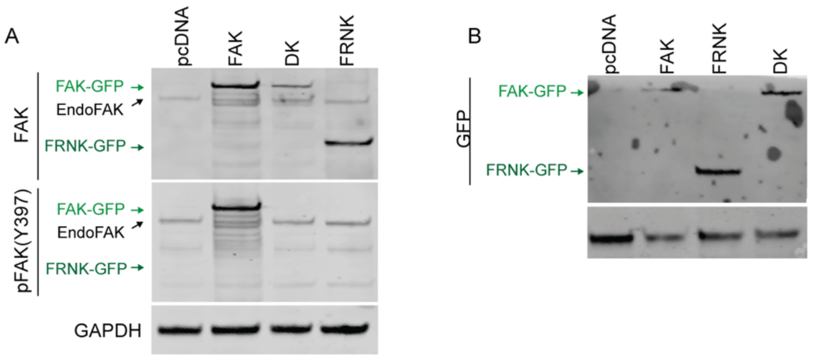

<u>Supplementary Figure 12:</u> Expression of FAK constructs in mouse SMCs. A. Western blot of FAK and pFAK (Y367) in mouse SMC transfected with empty pcDNA, FAK-GFP, DK-GFP, and FRNK-GFP. **B.** Western blot detecting GFP-fused constructs using a primary antibody against GFP.
