# Supplementary material for: Focal adhesion proteins confer smooth muscle anoikis resistance and protection against aortic aneurysm and dissection
Source: JCI Insight. 2026 Mar 24;11(9):e195291. doi: 10.1172/jci.insight.195291 (PMC13232016; doi:10.1172/jci.insight.195291)

Full unedited blot correspond to Figure 4D

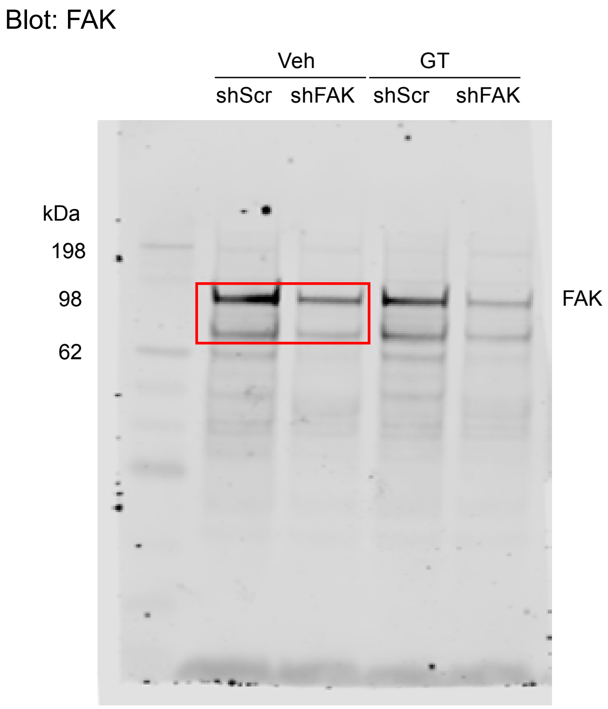

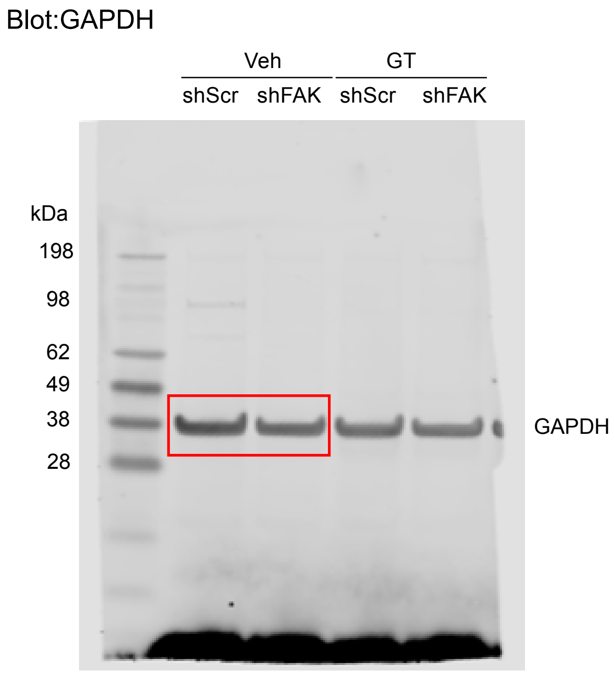

Full unedited blot correspond to Figure 4I

Blot: cleaved Caspase 3

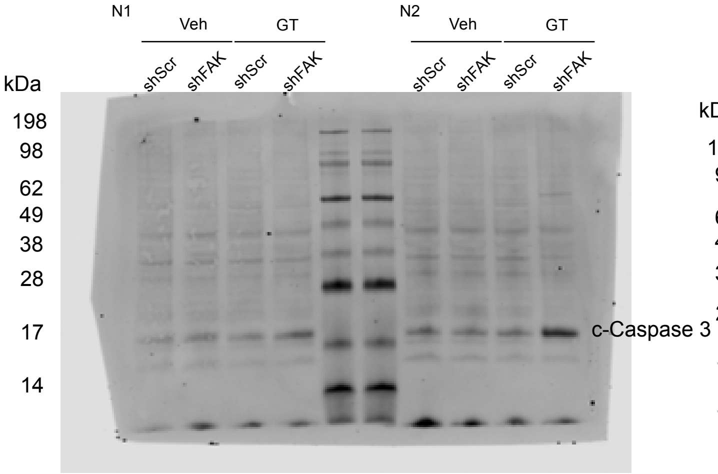

Blot: Caspase 3

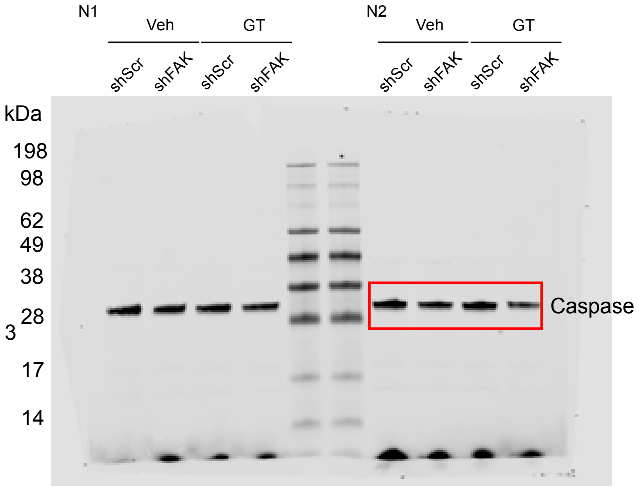

Co-exposure of Caspase 3 and c-Caspase 3

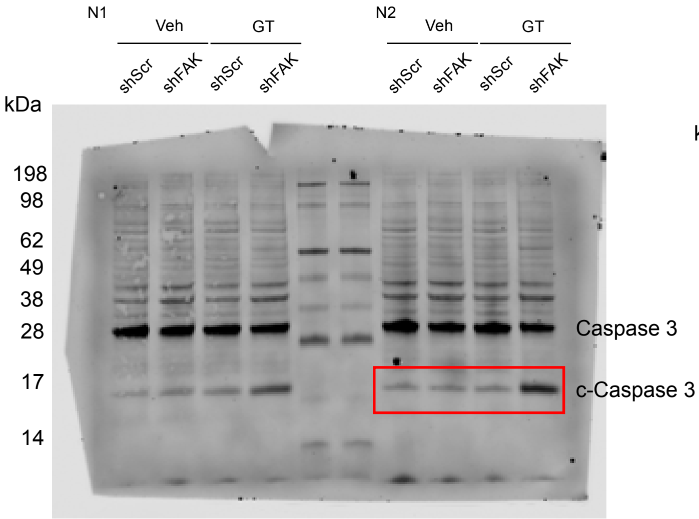

Blot: GAPDH

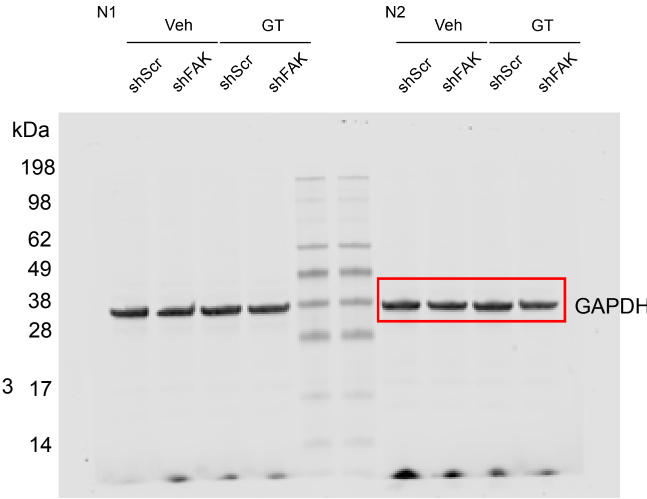

Full unedited blot correspond to Figure 5A

Blot:ILK

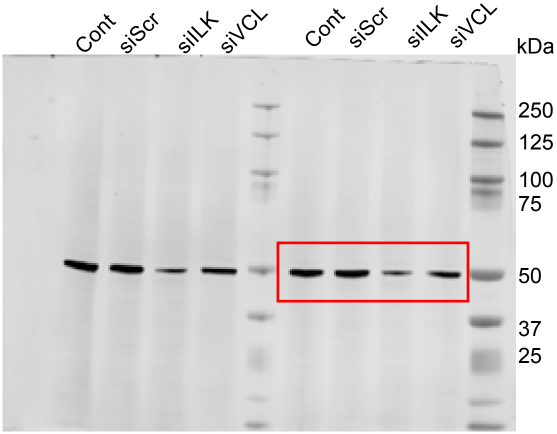

Blot:VCL

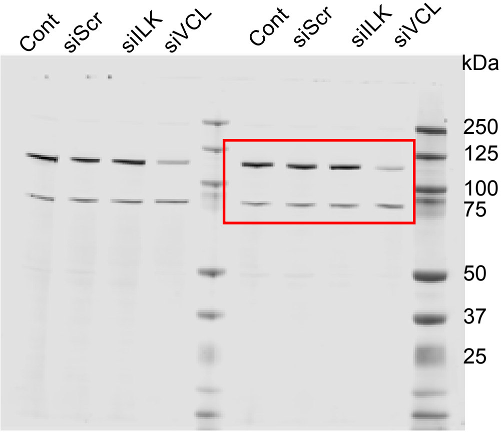

Blot:FAK

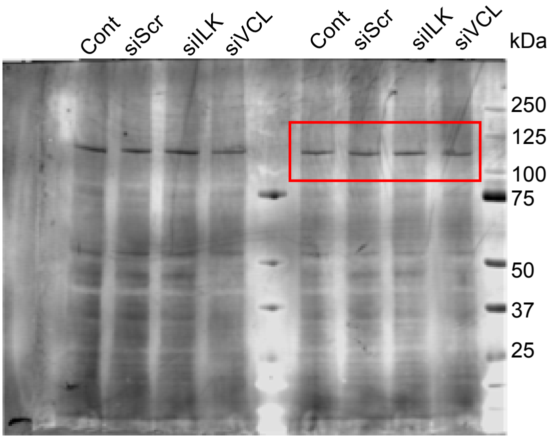

Blot:GAPDH

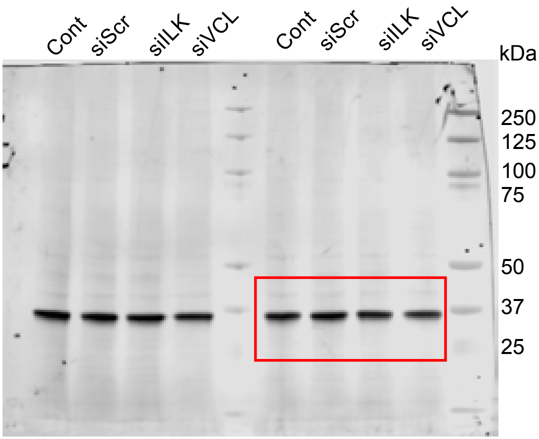

Cont: no transfection

Full unedited blot correspond to Figure 6C

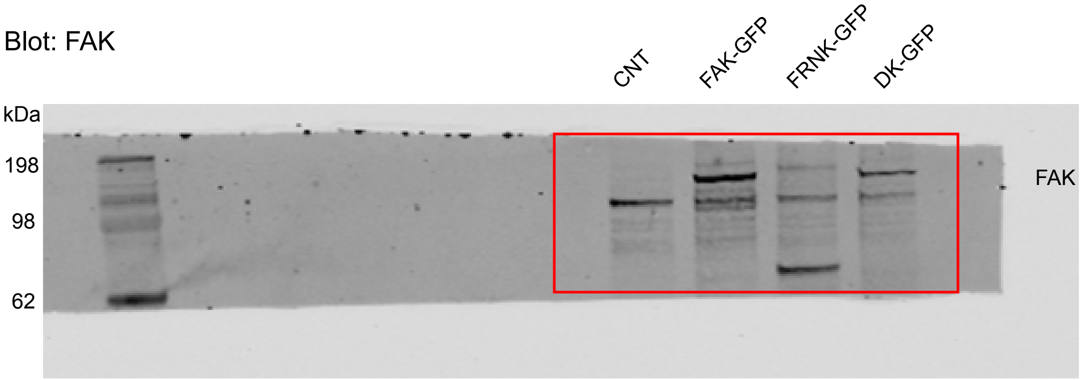

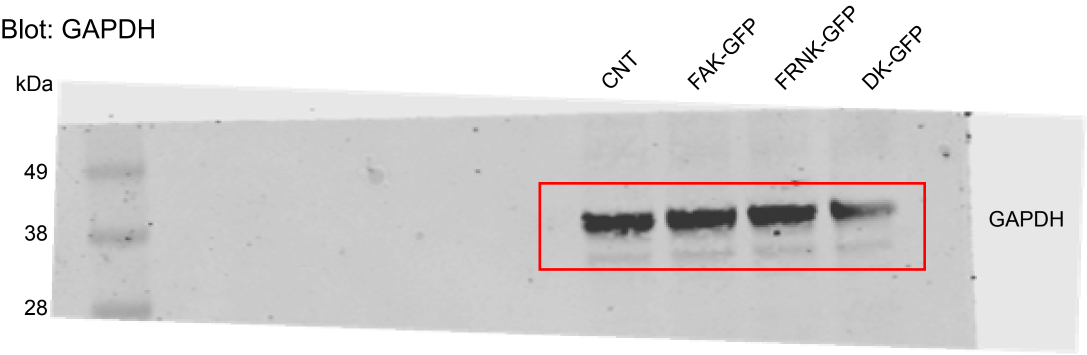

Blot:GAPDH

Full unedited blot correspond to Figure 7A

Blot:pFAK

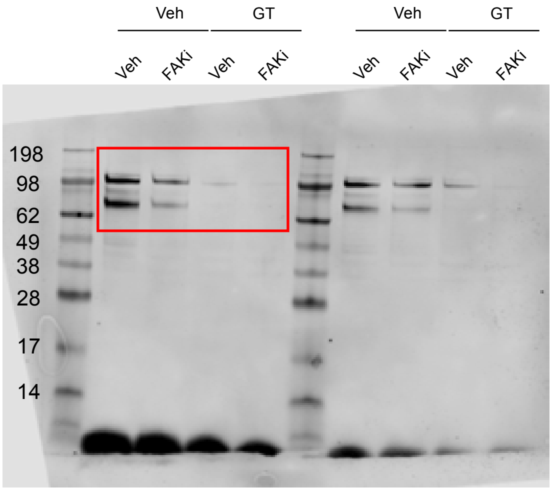

Blot:FAK

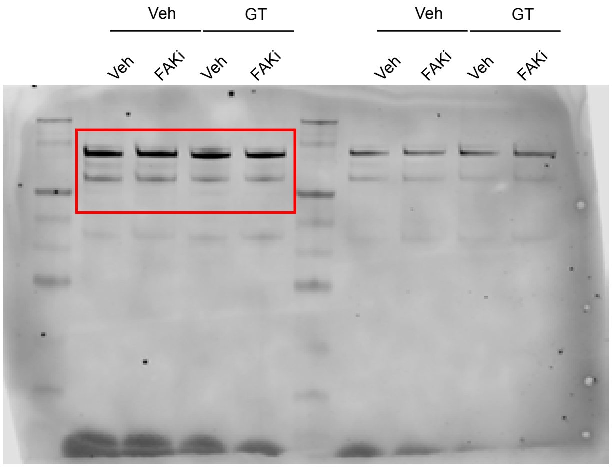

Blot:GAPDH

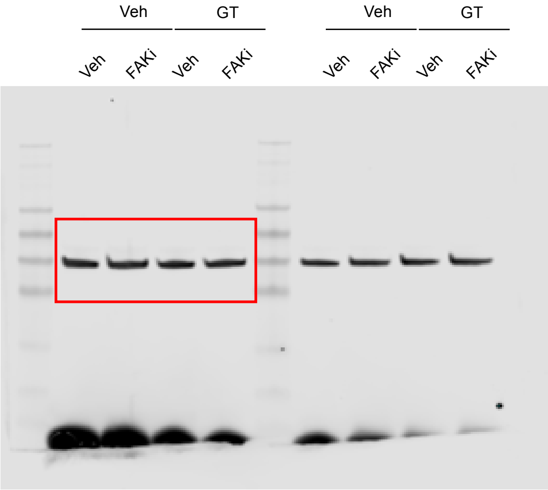

Full unedited blot correspond to Supplementary Figure 4C_left panel

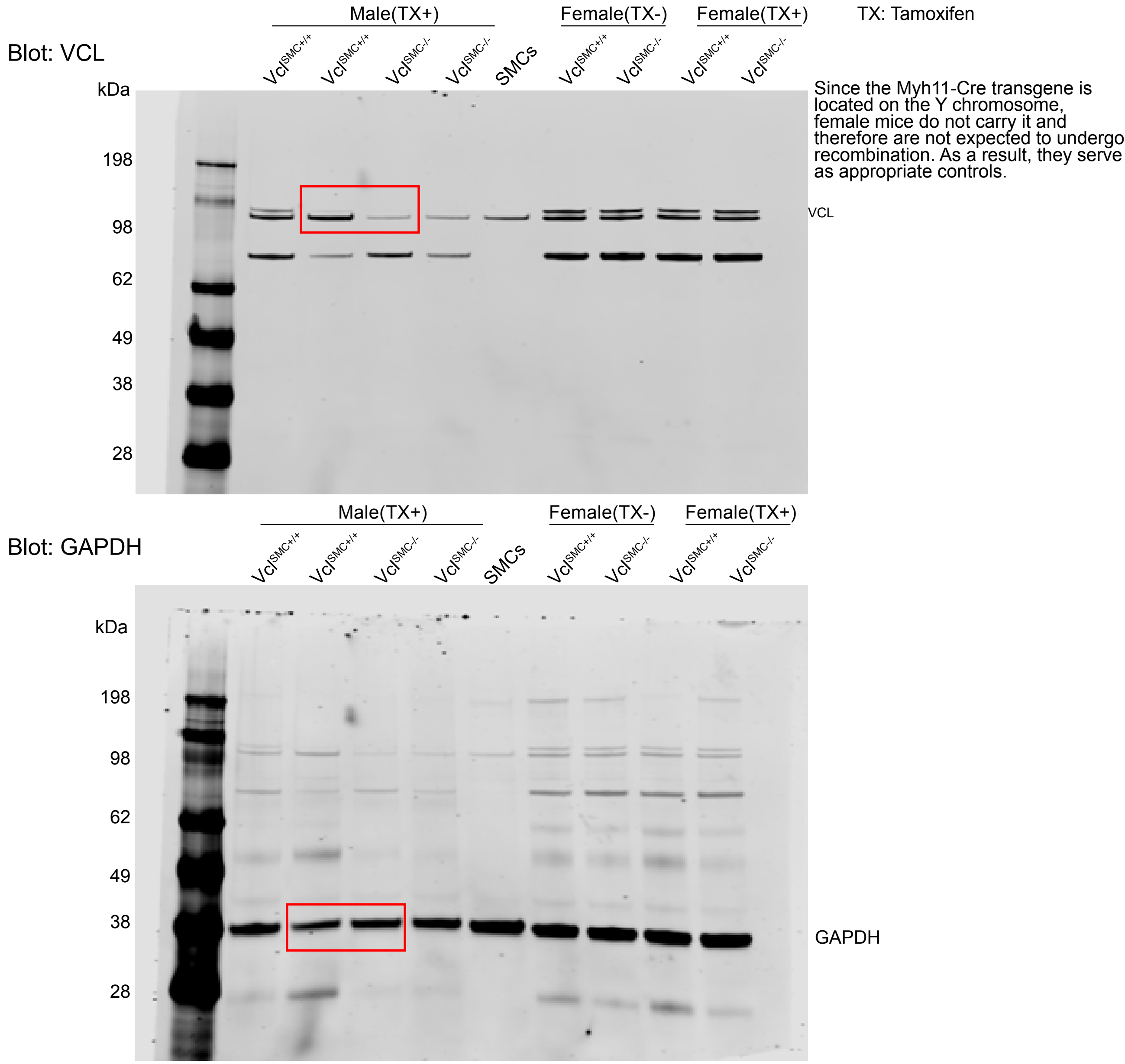

Full unedited blot correspond to Supplementary Figure 4C_middle panel

The third lane was excluded from analysis due to insufficient loading as indicated by weak GAPDH signal. The comparison between fourth lane (ILK knockout) and fifth lane (wildtype) shows similar trend to that observed between first and second lanes.

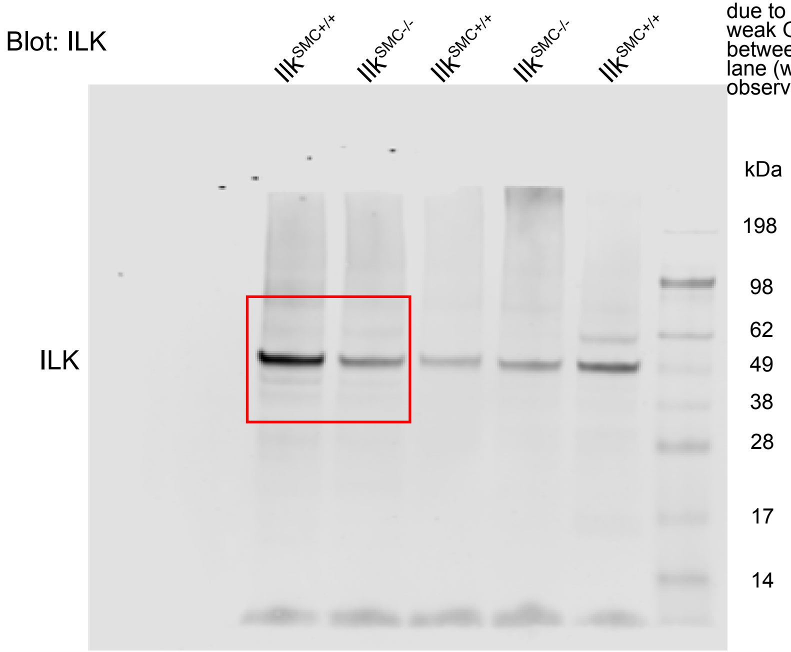

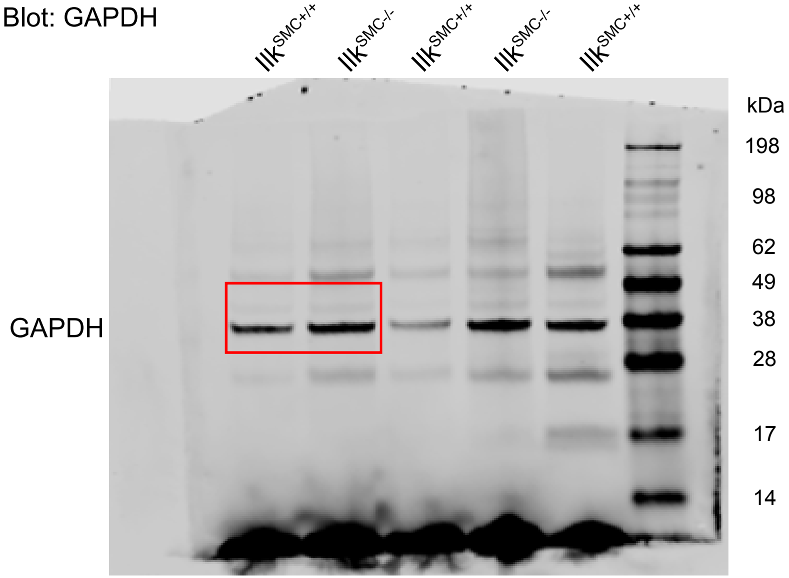

Full unedited blot correspond to Supplementary Figure 4C_right panel

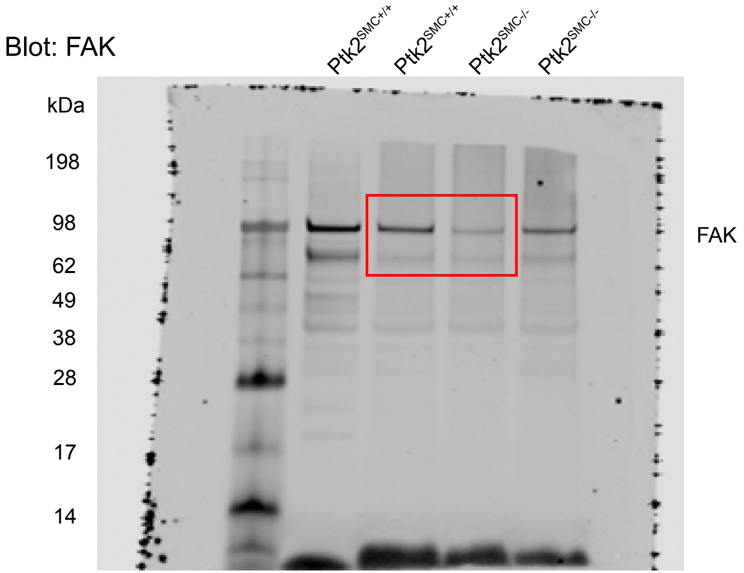

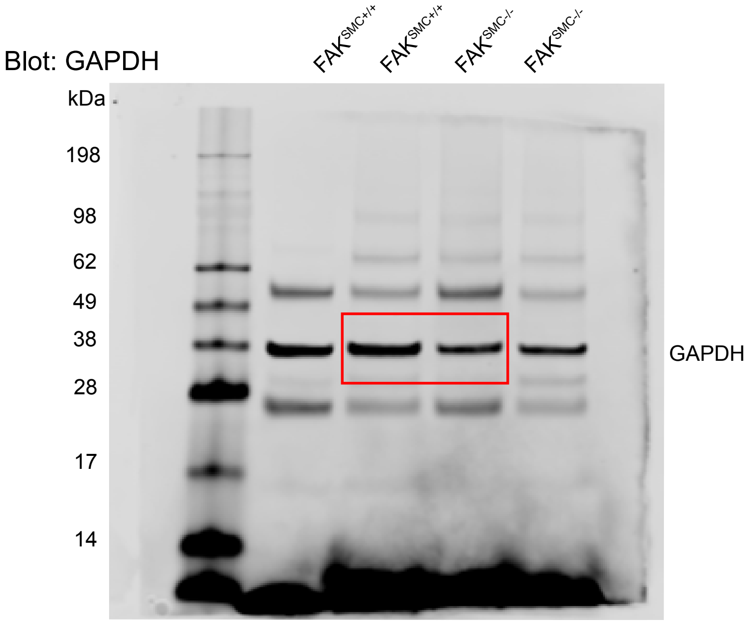

Full unedited blot correspond to Supplementary Figure 9A

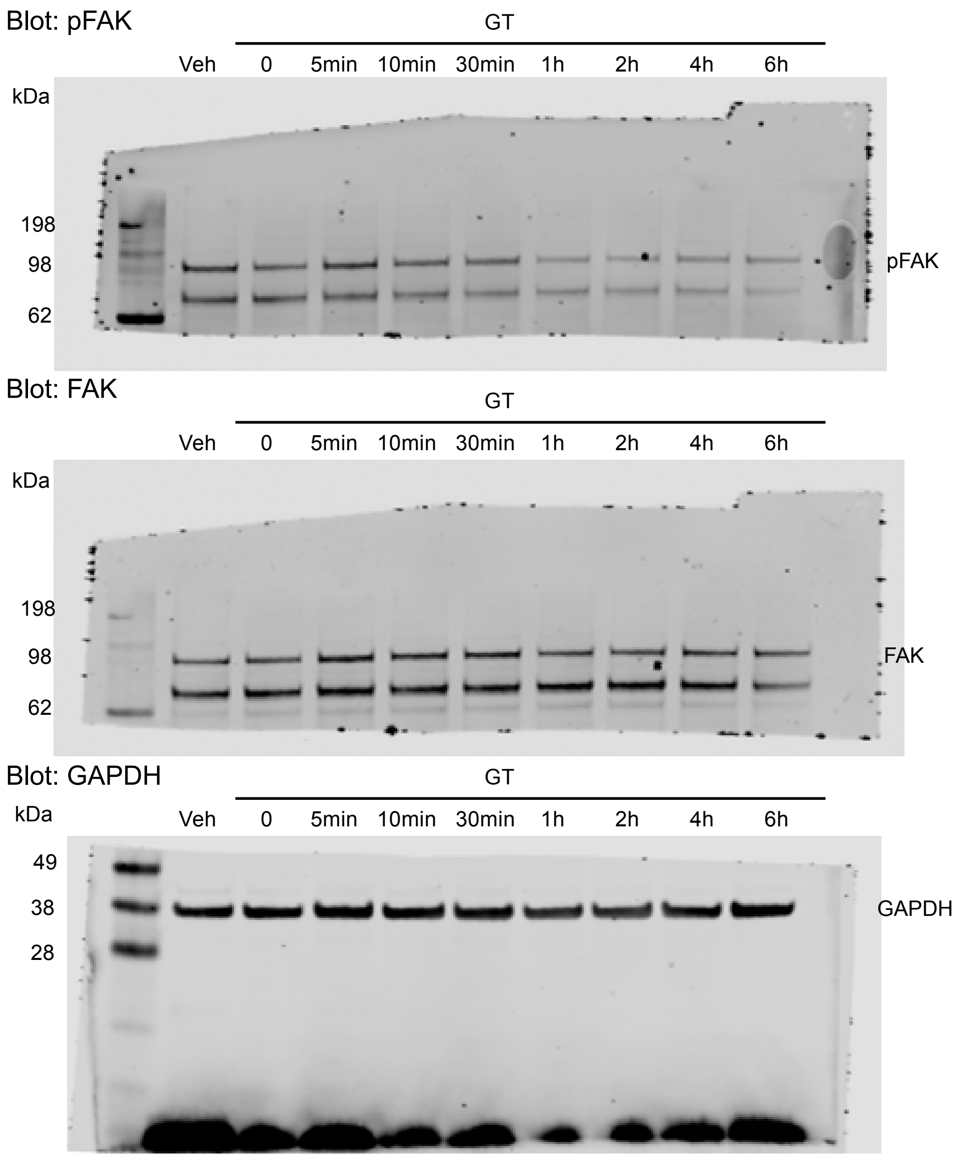

Full unedited blot correspond to Supplementary Figure 9C

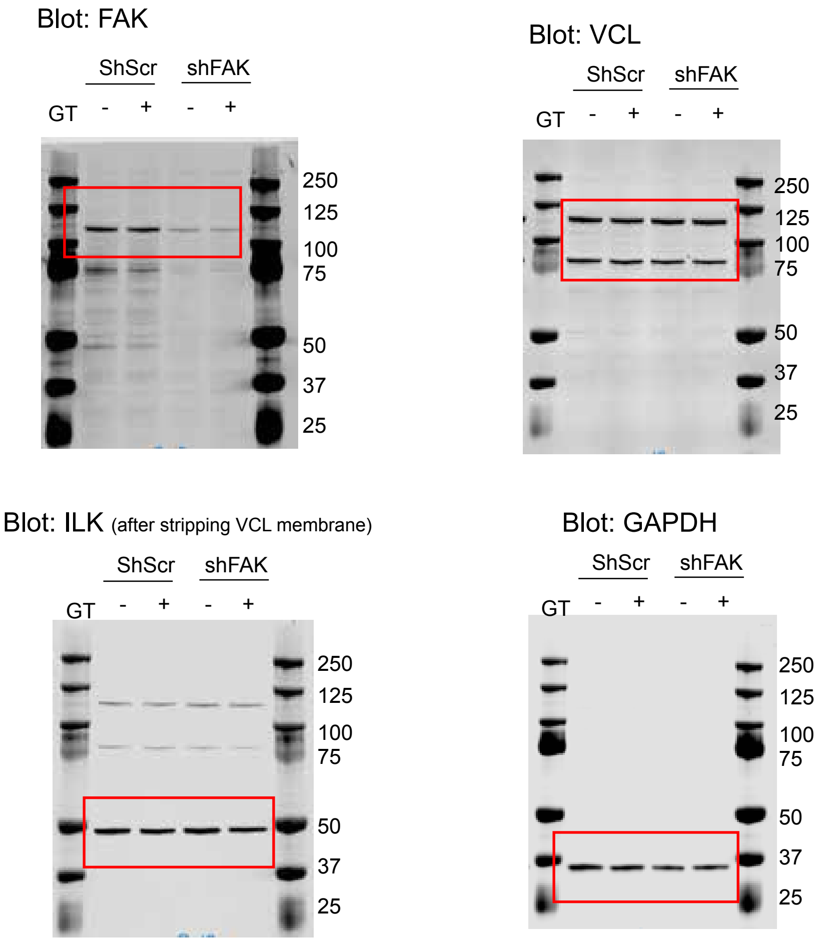

Full unedited blot correspond to Supplementary Figure 12A

Blot: pFAK

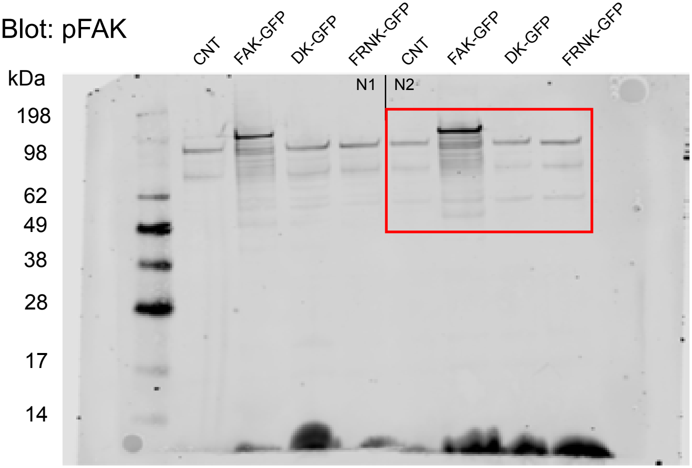

Blot: FAK

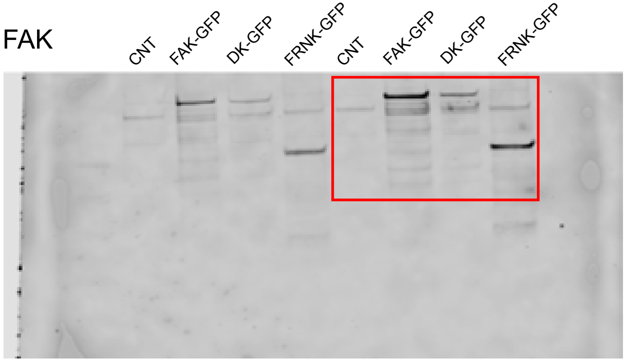

Blot: GAPDH

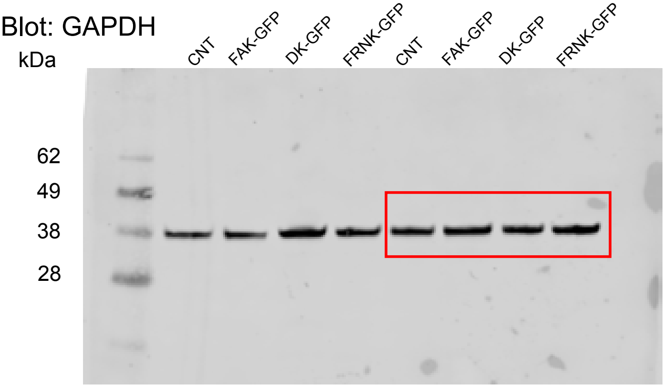

Full unedited blot correspond to Supplementary Figure 12B

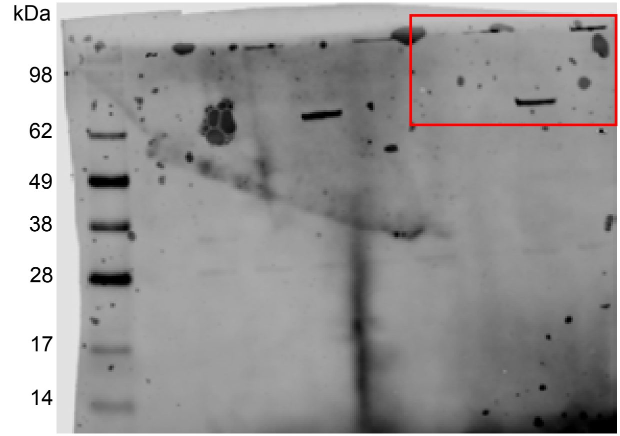

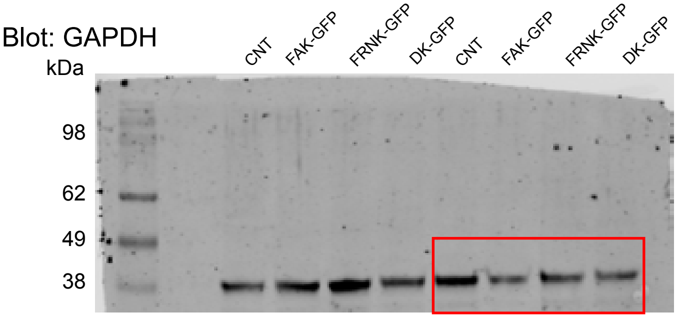

Supplement: Unedited blot and gel images [file jciinsight-11-195291-s024.pdf]
